# Immune‐related matrisomes are potential biomarkers to predict the prognosis and immune microenvironment of glioma patients

**DOI:** 10.1002/2211-5463.13541

**Published:** 2022-12-30

**Authors:** Hao Yu, Minjie Wang, Xuan Wang, Xiaobing Jiang

**Affiliations:** ^1^ Department of Neurosurgery, Union Hospital, Tongji Medical College Huazhong University of Science and Technology Wuhan China; ^2^ Department of Neurosurgery, Union Hospital, Tongji Medical College Wuhan China

**Keywords:** bioinformatics, ECM, glioma, immune microenvironment, matrisome, prognosis

## Abstract

The extracellular matrix (ECM) plays a vital role in the progression and metastasis of glioma and is an important part of the tumor microenvironment. The matrisome is composed of ECM components and related proteins. There have been several studies on the effects of matrisomes on the glioma immune microenvironment, but most of these studies were performed on individual glioma immune‐related matrisomes rather than integral analysis. Hence, an overall analysis of all potential immune‐related matrisomes in gliomas is needed. Here, we divided 667 glioma patients in The Cancer Genome Atlas (TCGA) database into low, moderate, and high immune infiltration groups. Immune‐related matrisomes differentially expressed among the three groups were analyzed, and a risk signature was established. Eight immune‐related matrisomes were screened, namely, LIF, LOX, MMP9, S100A4, SRPX2, SLIT1, SMOC1, and TIMP1. Kaplan–Meier analysis, operating characteristic curve analysis, and nomogram were constructed to analyze the relationships between risk signatures and the prognosis of glioma patients. The risk signature was significantly correlated with the overall survival of glioma patients. Both high‐ and low‐risk signatures were also associated with some immune checkpoints. In addition, analysis of somatic mutations and anti‐PD1/L1 immunotherapy responses in the high‐ and low‐risk groups showed that the high‐risk group had worse prognosis and a higher response to anti‐PD1/L1 immunotherapy. Our analysis of immune‐related matrisomes may improve understanding of the characteristics of the glioma immune microenvironment and provide direction for glioma immunotherapy development in the future.

## Graphical abstract

The extracellular matrix (ECM) plays a vital role in the progression and metastasis of glioma and is an important part of the tumor microenvironment. The matrisome is a collection of ECM molecules and related proteins. We screened immune‐related matrisomes, calculated risk scores, and established a risk model to evaluate the relationship between risk signatures and the prognosis of glioma patients.

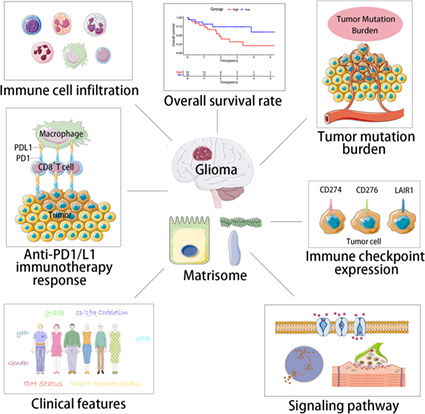

**Abbreviations**
BCCs: breast cancer cells
CGGA: Chinese Glioma Genome Atlas
CNS: central nervous system
ECM: extracellular matrix
EMT: epithelial mesenchymal transition
ESCC: esophageal squamous cell carcinoma
EVT: extravillous trophoblast
FPKM: fragments per kilobase per million
GBM: glioblastoma
GICs: glioma‐initiating cells
GM‐CSF: granulocyte macrophage colony stimulating factor
GO: Gene Ontology
HLA: human leukocyte antigen
IDH: isocitrate dehydrogenase
KEGG: Kyoto Encyclopedia of Genes and Genomes
KM: Kaplan–Meier
KPS: Karnofsky performance score
LASSO: least absolute shrinkage and selection operator
LGG: low‐grade glioma
LIF: leukemia inhibitory factor
LOX: lysyl oxidase
MDSCs: myeloid‐derived suppressor cells
MMP9: Matrix metalloproteinase‐9
OS: overall survival
PCR: polymerase chain reaction
ROC: operating characteristic curve
SMOC1: SPARC‐related modular calcium‐binding protein 1
ssGSEA: single‐sample gene set enrichment analysis
TCGA: The Cancer Genome Atlas
TIDE: tumor immune dysfunction and exclusion
TILs: tumor‐infiltrating lymphocytes
TMB: tumor mutation burden
TME: tumor microenvironment
Tregs: T cells regulatory

Glioblastoma (GBM) is the most prevalent and aggressive malignant tumor of the central nervous system (CNS), accounting for 14.6% of all CNS tumors [1]. Latest data show that approximately 100 000 incident cases of GBMs are diagnosed annually. Gliomas are differentiated from glial cells and are histologically classified as astrocytoma, oligodendroglioma, oligodendrocytoma, and ependymoma [2]. GBM has an unfavorable prognosis, with a median survival time of only 14–17 months and an average 2‐ and 3‐year survival rates of only 3.3% and 1.2%, respectively [3]. Although surgery combined with radiotherapy and chemotherapy has achieved good results in clinical trials, the recurrence rate is still very high [4]. This poor prognosis of glioma is attributed to several factors, such as the unique location, high heterogeneity, and tumor immunosuppressive microenvironment [5, 6]. Immunotherapy has unsatisfactory effects on glioma owing to the immune‐cold phenotype and unique immune microenvironment [7]. Therefore, there is an urgent need to identify improved indicators for the immune microenvironment of glioma.

The extracellular matrix (ECM), an important component of an organism, is a complex scaffold network composed of cross‐linked proteins such as collagen, non‐collagen, elastin, proteoglycan, and aminoglycan that supports surrounding cells [8]. ECM can provide a tissue scaffold to maintain the morphology and integrity of organs and is very important in cell development [9]. ECM proteins contain abundant signal molecules that control cell growth, polarity, shape, migration, and metabolic activity [10]. The collection of ECM molecules and related proteins is called a matrisome [11].

Extracellular matrix can also promote tumor growth, providing adhesion sites and cell signals to different cell groups, including cancer cells. ECM can also store many ECM‐modifying enzymes, ECM binding growth factors, and other ECM‐related proteins that assist with cell signaling of ECM proteins [12]. ECM is also involved in some pathological processes in the body, such as inflammation and tumors. Studies have shown that an increase in the ECM component is linked to tumor invasion and poor prognosis [13, 14]. The ECM plays a crucial role in tumor progression, invasion, and metastasis. For example, lysyl oxidase (LOX) is a monoamine oxidase associated with metastasis and adverse prognosis in gastric cancer [15]. Matrix metalloproteinase‐9 (MMP9) can act on the degradation of ECM and vascular remodeling, thus promoting tumor invasion [16].

Matrisomes are also involved in glioma development. The proteolytic activity of ECM is associated with invasion and metastasis of gliomas [17]. The immune microenvironment of glioma is composed of glioma cells, immune cells, and the ECM. Studies have shown that the ECM is related to the glioma immune microenvironment [18]. Thus, there have been several studies on the effects of matrisomes on the glioma immune microenvironment. However, most of these studies were performed on individual glioma immune‐related matrisomes rather than integral analysis.

Hence, an overall analysis of all potential immune‐related matrisomes in gliomas is needed. In addition, because of the important prognostic influence of immune infiltration in gliomas, it is essential to clarify whether immune‐related matrisomes have prognostic value. Thus, this study intended to analyze the expression profile of matrisomes in 667 patients with glioma, using the whole transcriptome data set from The Cancer Genome Atlas (TCGA) database.

## Materials and methods

### Data sources and clinical samples

Human matrisomes were collected from the Molecular Signatures Database V7.4. Meanwhile, the fragments per kilobase per million (FPKM) of transcriptome data, statistical data, and clinical features of patients with human low‐grade glioma (LGG) and GBM were extracted from the Cancer Genome Atlas Database (TCGA, https://cancergenome.nih.gov/) [19]. Patient characteristics included the total number of patients (*n* = 667), gender, age, grade, isocitrate dehydrogenase (IDH) status, Karnofsky performance score (KPS), chemotherapy, radiotherapy, and MGMT promoter methylation status. Verified FPKM transcriptome data and patient clinical characteristics (*n* = 970) were extracted from the Chinese Glioma Genome Atlas (CGGA) database (http://www.cgga.org.cn/) [20]. We also verified it by GEO database under accession number GSE150604 (*n* = 239). We have drawn a flow chart (Fig. S8) more intuitively to display the research ideas.

In addition, brain tissue samples of 26 patients (Table S6) with GBM and 18 patients with paracancerous lesions were obtained from Union Hospital, Tongji Medical College, Huazhong University of Science and Technology, Wuhan, China. The study was conducted according to the guidelines of the Declaration of Helsinki, and approved by the Ethics Committee of Tongji Medical College, Huazhong University of Science and Technology (protocol code: [2019]IEC(S742), date of approval: MAR 4th 2019). All patients provided written informed consent for the use of their samples and data.

### Generation and verification of immune grouping

We used single‐sample gene set enrichment analysis (ssGSEA) and r package “gsva” to analyze the level of immune infiltration in glioma samples. By utilizing unsupervised hierarchical clustering algorithm, the glioma patients were classified into high immune cell infiltration cluster, middle immune cell infiltration cluster and low immune cell infiltration cluster according to the level of immune cell infiltration using “hclust” (r package). To verify the correctness of clustering, immune cell expression in the three groups was calculated using the CIBERSORT deconvolution algorithm, which is a method that can explore the stromal and immune cell proportion using gene expression profiles, and a heatmap was generated. The differences among the three groups were verified by stromal score, immune score, estimated score, tumor purity, human leukocyte antigen (HLA) expression level, and immune cell composition.

### Screening of immune‐related matrisomes

We divided the TCGA gene expression profile data of glioma patients into three groups: low, medium, and high immune cell infiltration. Taking |log_2_FC| > 1, *P* value < 0.05 as the standard, the differentially expressed matrisomes between the low and moderate immune cell infiltration groups and between the moderate and high immune cell infiltration groups were analyzed using “edger” package. Using online Venn analysis, overlapping matrisomes between the above two groups of differentially expressed matrisomes were selected to screen immune‐related matrisomes.

### Establishing a risk signature based on immune‐related prognostic matrisomes

The relationship between immune‐related matrisome expression and overall survival (OS) of glioma patients was analyzed using univariate Cox regression. In univariate Cox analysis, a *P*‐value < 0.05 was considered statistically significant. Least absolute shrinkage and selection operator (LASSO) regression was used to screen the applicable combination of immune‐related prognostic matrisomes, and a risk signature was constructed. The candidate genes were divided into two groups: risk type (HR > 1) and protective type (0, < 1). A prognostic risk score equation was then established based on the results of LASSO analysis, and a linear combination of expression level and regression coefficient weighting was used. The risk score equation was as follows:

$$\text{risk score}=\text{matrisome}\;\left(1\right)\times \beta \left(1\right)\;\text{expression}+\text{matrisome}\;\left(2\right)\times \beta \left(2\right)+\cdots \cdots +\text{Matrisome}\;n\times \beta \left(n\right).$$

Kaplan–Meier (KM) curves and time‐dependent receiver operating characteristic (ROC) curves were then generated based on the median risk score to verify the prognostic significance of risk signature [21]. A total of 667 patients with glioma were divided into the high‐risk and low‐risk subgroups (Table S1). The effectiveness of the risk signature was further verified using the CGGA data set comprising 970 glioma patients (Table S2) and GEO database under accession number GSE150604 comprising 239 glioma patients (Table S10).

### Establishing and assessing the nomogram

To improve the clinical value of the risk signature in the clinic, we conducted univariate and multivariate Cox regression analyses to determine the relationship between factors (gender, age, grade, IDH status, 1p/19q codeletion, Karnofsky performance score, MGMT promoter status, history of radiotherapy and chemotherapy, and risk score) and OS. The nomogram was established using the TCGA database and verified using the CGGA database. The prognostic predictive accuracy of the nomogram was demonstrated using calibration curves. These analyses were performed using the r package “rms.” ROC curves were used to evaluate the prognostic capacity of the nomogram and other predictors (risk score, age, grade, IDH mutation status, and 1p/19q codeletion) for 1‐, 3‐, and 5‐year OS of patients with glioma.

### Functional enrichment analysis

To further explore the eight immune‐related matrisomes, we screened differentially expressed genes between the high‐ and low‐risk subgroups using r software package “edger.” The functional enrichment of the differentially expressed genes was then analyzed with the Gene Ontology (GO) enrichment analysis and Kyoto Encyclopedia of Genes and Genomes (KEGG) using the r software package “ggpolt2,” with |log_2_FC| > 1 and *P* value < 0.05 as the standard.

### Immunohistochemistry

The paraffin‐embedded tissues of 16 patients with GBM and 12 patients with paracancerous brain tissue were cut into 5 μm‐thick sections and stained with hematoxylin. The sections were incubated with anti‐leukemia inhibitory factor (LIF), LOX, MMP9, S100A4, SRPX2, SLIT1, SMOC1, and TIMP1 primary antibodies at 4 °C; washed with phosphate buffer three times; and incubated with secondary antibody at room temperature for 30 min.

We used 3,3‐diaminobenzidine (DAB; 1 : 50) as the chromogenic substrate and counterstained with hematoxylin [22]. The staining was evaluated by two independent pathologists who were blinded to the patients' clinical data. The tissue sections were scored according to the percentage of staining positive cells as follows: 0, 0%; 1, 1–10%; 2, 11–25%; 3, 26–50%; 4, 51–70%; and 5, 71–100%. Meanwhile, staining intensity was scored as 0 for negative staining; 1, weak staining; 2, moderate staining; and 3, strong staining. The final staining score was calculated as the product of staining intensity and percentage of positive cells. The samples with a final staining score of < 3 and ≥ 3 were divided into the negative and positive expression groups, respectively.

### Real‐time quantitative reverse transcriptase‐polymerase chain reaction

Total RNA was extracted from 10 glioma and 6 adjacent brain tissue samples using TRIzol reagent (Thermo Fisher Scientific, Waltham, MA, USA). A reverse transcription kit (Vazyme, Wuhan, China) was used to reverse transcribe cDNA, and then real‐time polymerase chain reaction (PCR) was performed with SYBR Green real‐time PCR kit, with GAPDH as the internal reference control. The PCR cycle conditions were as follows: 95 °C for 2 min, then 94 °C for 20 s, 58 °C for 20 s, and 72 °C for 30 s, for a total of 40 cycles. All RT‐qPCR reactions were independently performed three times. The tissue samples and primer sequences of the matrisomes are shown in Tables S3 and S4.

### Tumor mutation burden and anti‐PD1/L1 therapy of risk groups

Somatic mutation data were obtained from TCGA database. Somatic mutations mainly included frameshift mutations, non‐synonymous mutations, non‐silent mutations, frameshift mutations, and deletion mutations. The tumor mutation burden (TMB) as the number of somatic mutations per megabyte. We used the r package “maftools” to analyze the mutation profiles [23]. Meanwhile, tumor immune dysfunction and exclusion (TIDE; http://tide.dfci.harvard.edu) and immune cell abundance identifier (ImmuCellAI; http://bioinfo.life.hust.edu.cn/ImmuCellAI) algorithms were used to analyze the potential response to PD1/L1 therapy.

### Statistical analysis

The ROC curve was generated to measure the accuracy of survival prediction, using the r package “survivalroc.” Student's *t*‐test was used to compare the risk scores among subgroups based on clinical characteristics. The Wilcoxon test was used to compare the immune infiltration data between the risk groups. GSEA was used for the functional analysis [24]. All statistical analyses were performed using the r 4.0.2. Statistical significance was defined as a two‐tailed *P*‐value < 0.05 (***P < 0.001; **P < 0.01; and *P < 0.05). All pictures were drawn using the r language.

## Results

### Identification of immune‐related matrisomes in glioma patients

A total of 667 low‐grade glioma (LGG) and GBM samples were extracted from TCGA database. The clinical characteristics of the patients are summarized in Table 1.

**Table 1:** The clinical features of glioma patients in TCGA and CGGA database. KPS, Karnofsky Performance Score.

Characteristic	TCGA (*n* = 667)	CGGA (*n* = 970)
Age (years)		
≤ 60	472	877
> 60	195	93
Gender		
Female	255	399
Male	356	571
NA	56	0
WHO grade		
II	215	270
III	236	322
IV	160	374
NA	56	4
IDH status		
Mutant	401	500
Wild type	257	421
NA	9	49
1p19q codeletion		
Codel	166	199
Non‐codel	495	697
NA	6	74
KPS		
≤ 80	193	–
> 80	217	–
NA	257	–
MGMT promoter status		
Methylated	472	456
Unmethylated	161	361
NA	34	153
TERT promoter status		
Mutant	153	–
Wild type	202	–
NA	312	–
TMZ chemotherapy		
Yes	393	670
No	10	266
NA	264	34
Radiotherapy		
Yes	388	742
No	178	193
NA	101	35

**Fig. 1:**
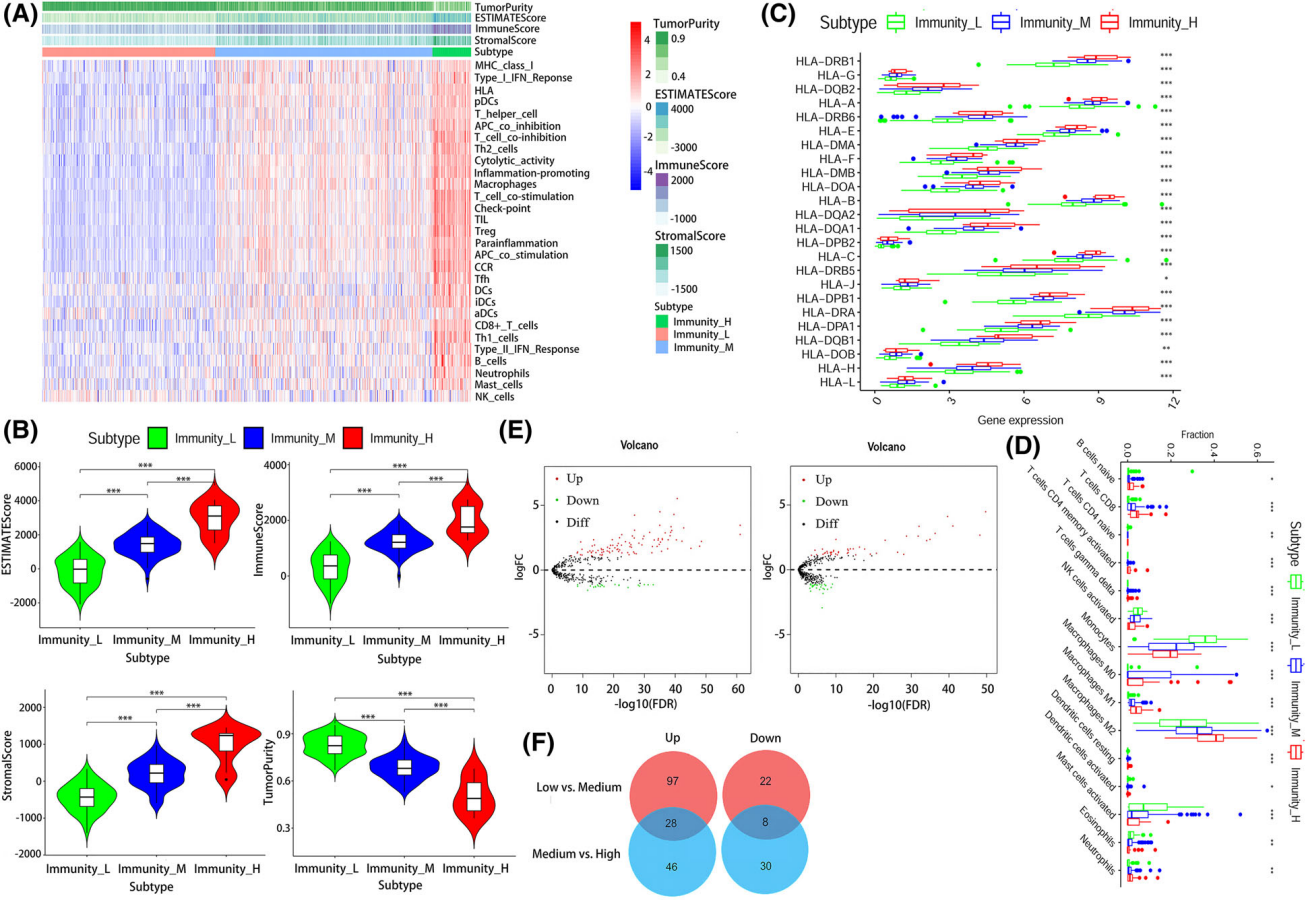
Characteristics of immune grouping and screening of immune‐related matrisomes. (A) A total of 667 glioma samples were divided into three groups based on immune cell infiltration: low infiltration group (immunity_L, *n* = 269), medium infiltration group (immunity_M, *n* = 338), and high infiltration group (immunity_H, *n* = 61). (B) ESTIMATE score, immune score, stromal score, and tumor purity of immune groups. (C) HLA expression of the three immune groups. (D) The immune infiltration of different groups. (E) and (F) are differentially expressed matrisomes and intersection Venn diagrams of the three immune groups.

Of these, 269, 338, and 61 samples were categorized into the low infiltration (immunity_L), moderate infiltration (immunity_M), and high infiltration (immunity_H) groups, respectively, using the unsupervised hierarchical clustering algorithm (Fig. 1A). As seen in Fig. 1B–D, there were notable differences in immune score, stromal score, and estimate score among the three groups, and the score increased gradually from the low immune group to the high immune infiltration group (*P* < 0.001). Tumor purity was also significantly different, increasing from the high infiltration group to the low infiltration group (*P* < 0.001).

In addition, HLA expression and the types of immune cell infiltration in the high infilitration group were higher than those in the other two groups (*P* < 0.001). For differentially expressed matrisomes, 119 were differentially expressed between the low and moderate infiltration group; of these 97 and 22 matrisomes were upregulated and downregulated, respectively (Tables S7 and S8). Meanwhile, 76 matrisomes were differentially expressed between the moderate and high infiltration groups; of these, 46 and 30 were upregulated and downregulated, respectively (Fig. 1E,F).

By intersecting with the above results, 36 differentially expressed matrisomes among the three groups were further screened by Venn analysis (Table S9) among them, 28 and 8 were upregulated and downregulated, respectively.

### Prognostic value of the risk signature

**Fig. 2:**
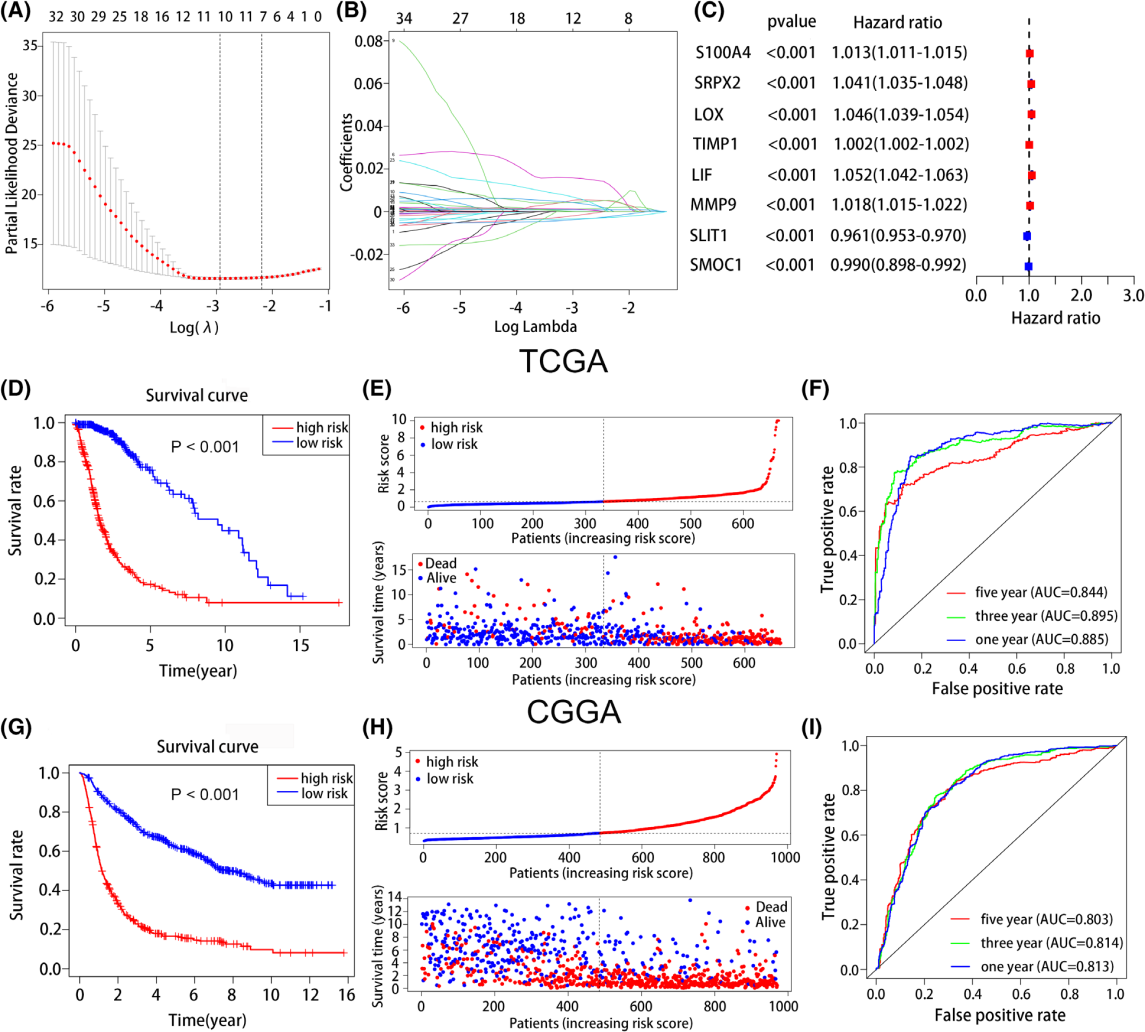
Establish and analyze immune‐related matrisomes risk signatures for the prognosis of glioma. (A, B) LASSO regression analysis verified the prognosis of eight immune‐related matrisomes. (C) Univariable Cox regression of eight immune‐related matrisomes in TCGA database. (D) The KM curve showed that the overall survival rate in the high‐risk group was worse than that in the low‐risk group in TCGA database. (E) The risk curve and scatter plot of high and low group in TCGA database. (F) The AUCs of the 1‐, 3‐, and 5‐year survival rates in TCGA database. (G) The KM curve indicated that the overall survival rate in the high‐risk group was worse than that in the low‐risk group in CGGA database. (H) The risk curve and scatter plot of high and low group in CGGA database. (I) The AUCs of the 1‐, 3‐, and 5‐year survival rates in CGGA database.

In total, eight immune‐related matrisomes were identified to have prognostic value, namely, LIF, LOX, MMP9, S100A4, SRPX2, SLITI1, SMOC1, and TIMP1 (Fig. 2A–C). The univariate analysis results showed that among the eight immune‐related matisomes we screened, LIF, LOX, MMP9, S100A4, SRPX2, and TIMP1 were considered as risk effectors with HR > 1 both in TCGA and CGGA databases. However, the HR values of the other two immune‐related matrisomes SMOC1 and SLITI1 in TCGA database were 0.990 and 0.961, close to 1 but less than 1, while the HR values of SMOC1 and SLIT1 in CGGA database were 0.804 and 0.755, both less than 1, which can be considered as protective effectors. The survival analysis of their prognostic impact was shown in Fig. S1. According to median score, 315 and 352 patients were classified into the high‐ and low‐risk groups. LIF, LOX, MMP9, S100A4, SRPX2, and TIMP1 were upregulated in the high‐risk group, whereas SLIT1 and SMOC1 were downregulated in the low‐risk group. The OS rate was significantly worse in the high‐risk group than in the low‐risk group (*P* < 0.001) (Fig. 2D), demonstrating that the risk score had good prognostic value.

The risk curve and scatter plot showed that the mortality rate was higher in the high‐risk group than in the low‐risk group (Fig. 2E). On ROC curve analysis to verify the predictive significance of the risk signatures, the AUCs for predicting 1‐, 3‐, and 5‐year survival rates were 0.885, 0.895, and 0.844, respectively, in TCGA database (Fig. 2F). Further verification using the CGGA database showed AUCs of 0.813, 0.814, and 0.803 for predicting the 1‐, 3‐, and 5‐year survival rates, respectively (Fig. 2G–I). We also utilized GEO database under accession number GSE150604 to verify the predictive significance of the risk signatures (Fig. S5A–C).

### Association between risk signature and clinical parameters

**Fig. 3:**
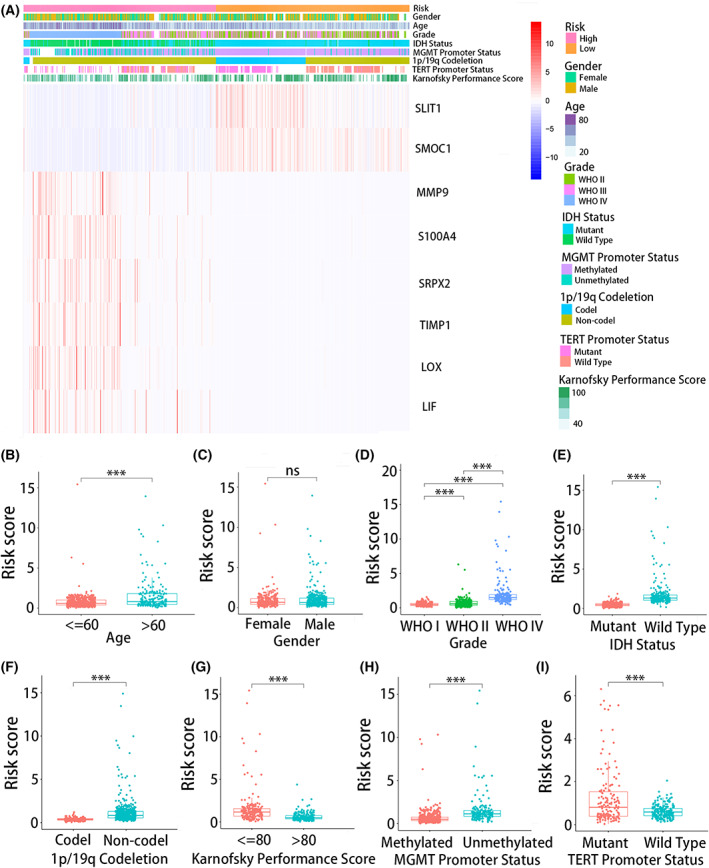
Relationship between risk signature and clinical features of glioma in TCGA database. (A) The heatmap showed the relationship between high‐ and low‐risk groups and clinical features of glioma patients. Risk scores of high‐ and low‐groups of (B) age, (C) gender, (D) WHO grade, (E) IDH mutation status, (F) 1p/19q codeletion, (G) Karnofsky performance score, (H) MGMT promoter methylation status and (I) TERT promoter status. (B–I) were performed in triplicate, and the *t* test was performed. *P < 0.05, **P < 0.01, and ***P < 0.001.

The heatmap showed the relationship between high‐ and low‐risk groups and clinical features of glioma patients, such as gender, age, WHO grade, IDH mutation status, KPS, 1p/19q codeletion, TERT promoter status, and MGMT promoter methylation status, in TCGA database (Fig. 3A). There were more elderly patients in the high‐risk group, and patients aged < 60 years had significantly lower risks of mortality than did patients aged > 60 years (*P* < 0.001) (Fig. 3B). Patients with KPS > 80 had a significantly lower risk score than those with a KPS < 80 (*P* < 0.001) (Fig. 3G). There was no significant difference in the distribution of gender between the high‐risk and low‐risk groups (Fig. 3C).

Meanwhile, there were more high WHO grade patients in the high‐risk group and more low WHO grade patients in the low‐risk group (*P* < 0.001) (Fig. 3D). For IDH mutation status, IDH wild‐type patients tended to be included in the high‐risk group, while IDH‐mutant patients tended to belong to the low‐risk group (*P* < 0.001) (Fig. 3E). In addition, 1p/19q non‐codeletion and MGMT promoter unmethylation were more frequent in the high‐risk group (*P* < 0.001) (Fig. 3F,H). For TERT promoter status, patients with TERT promoter wild type had lower risk score than did patients with TERT promoter mutations (*P* < 0.001) (Fig. 3I). The clinical characteristics of glioma patients in the high‐ and low‐risk groups in the CGGA database and GEO data set were shown in Figs S2 and S6.

### Nomogram performance for predicting 1‐, 3‐ and 5‐year survival rates

**Fig. 4:**
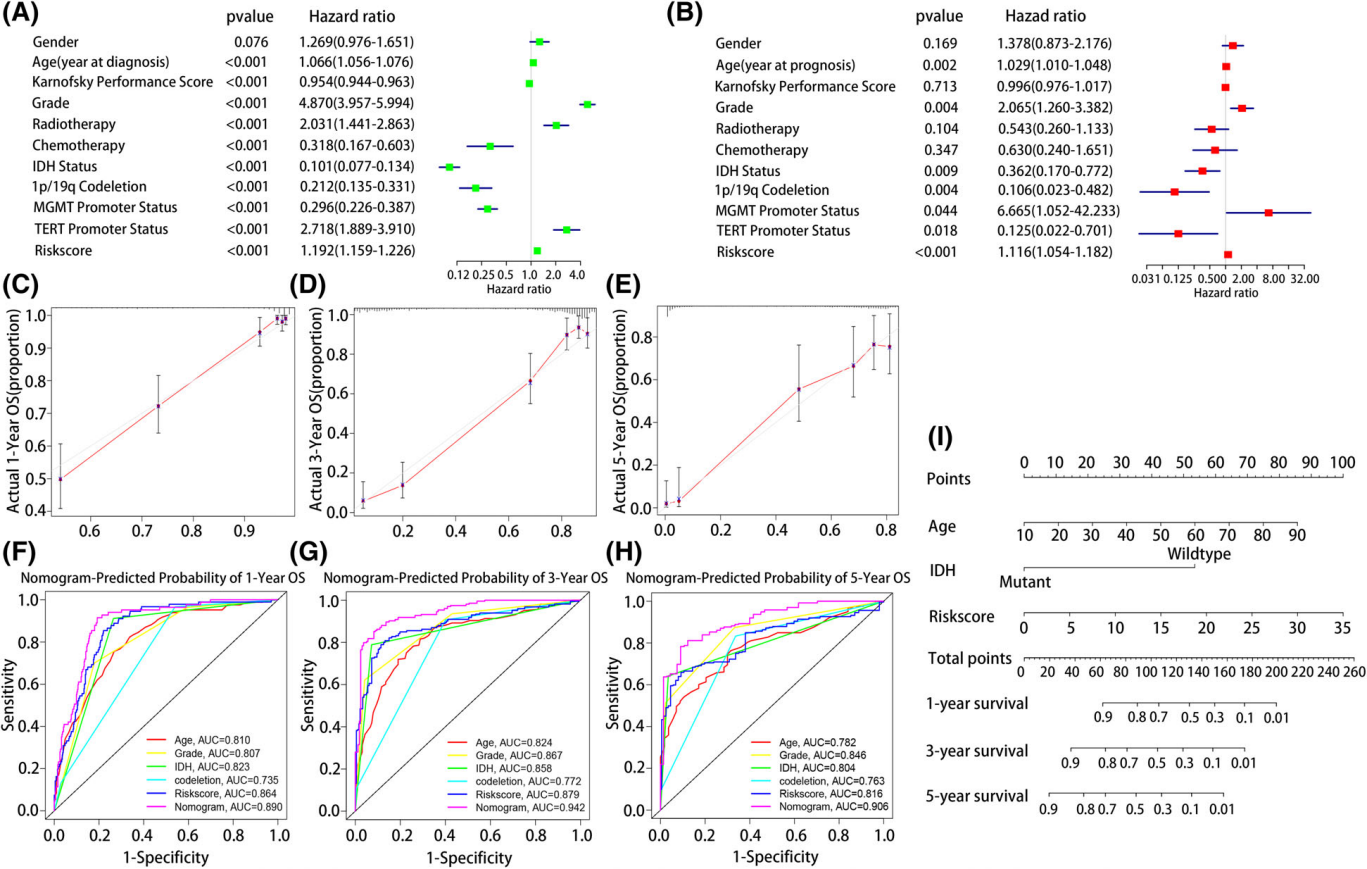
A nomogram based on risk score and some clinical characteristics was established. (A) Univariate and (B) multivariate Cox regression analyses were used to verify the prognostic value of risk signature. (C–E) Calibration plots for predicting patient 1‐, 3‐ and 5‐year OS. (F–H) ROC curves were used to evaluate the predictive ability of the nomogram and other predictors (1‐, 3‐ and 5‐year). (I) The nomogram for predicting proportion of patients with 1‐, 3‐ and 5‐year OS.

The results of univariate and multivariate Cox analyses showed that the risk signature can be used as an independent prognostic factor in patients with glioma in TCGA database (Fig. 4A,B), CGGA database (Fig. S3A,B), and GEO data set (Fig. S5D,E). For practical application, a nomogram was established using the 667 glioma patients of TCGA database to predict the 1‐, 3‐, and 5‐year OS (Fig. 4I), and the predictive performance was verified from 970 glioma patients in the CGGA database (Fig. S3). The predictors included risk score, age, grade, IDH mutation, and 1p/19q codeletion status. The actual probabilities of 1‐, 3‐, and 5‐year OS in the TCGA cohort (Fig. 4C–E), the CGGA cohort (Fig. S3C–E) and GEO data set (Fig. S7A–C) were consistent with those predicted by the nomogram. We used decision curve analysis to verify the diagnostic capacity of the nomogram and other predictors (age, grade, IDH mutation status, 1p/19q codeletion and riskscore), as shown in Fig. S9. The results show that nomogram can be used as a predictor. We also used Schoenfeld residual graph method to test the proportional risk hypothesis of nomogram. The result showed that the Schoenfeld residual chart has no time‐related change trend, which conformed to the proportional hazards assumption (Fig. S10).

In addition, time‐dependent ROC curves to evaluate the predictive capability of the nomogram and other predictors (risk score, age, grade, IDH status, and 1p/19q codeletion) showed AUCs of 0.890, 0.942, and 0.906 for TCGA (Fig. 4F–H), respectively, and 0.788, 0.842, and 0.843, for the CGGA database (Fig. S3F–H), respectively. In GEO data set, time‐dependent ROC curves to evaluate the predictive capability of the nomogram and other predictors (risk score, age, grade, Karnofsky Performance Score, MGMT promoter methylation status, and Mini‐mental State Examination) showed AUCs of 0.907, 0.887, and 0.936. These results support that the nomogram is more reliable and accurate predictor of prognosis.

### Correlation between risk signature and immune infiltration of glioma microenvironment

**Fig. 5:**
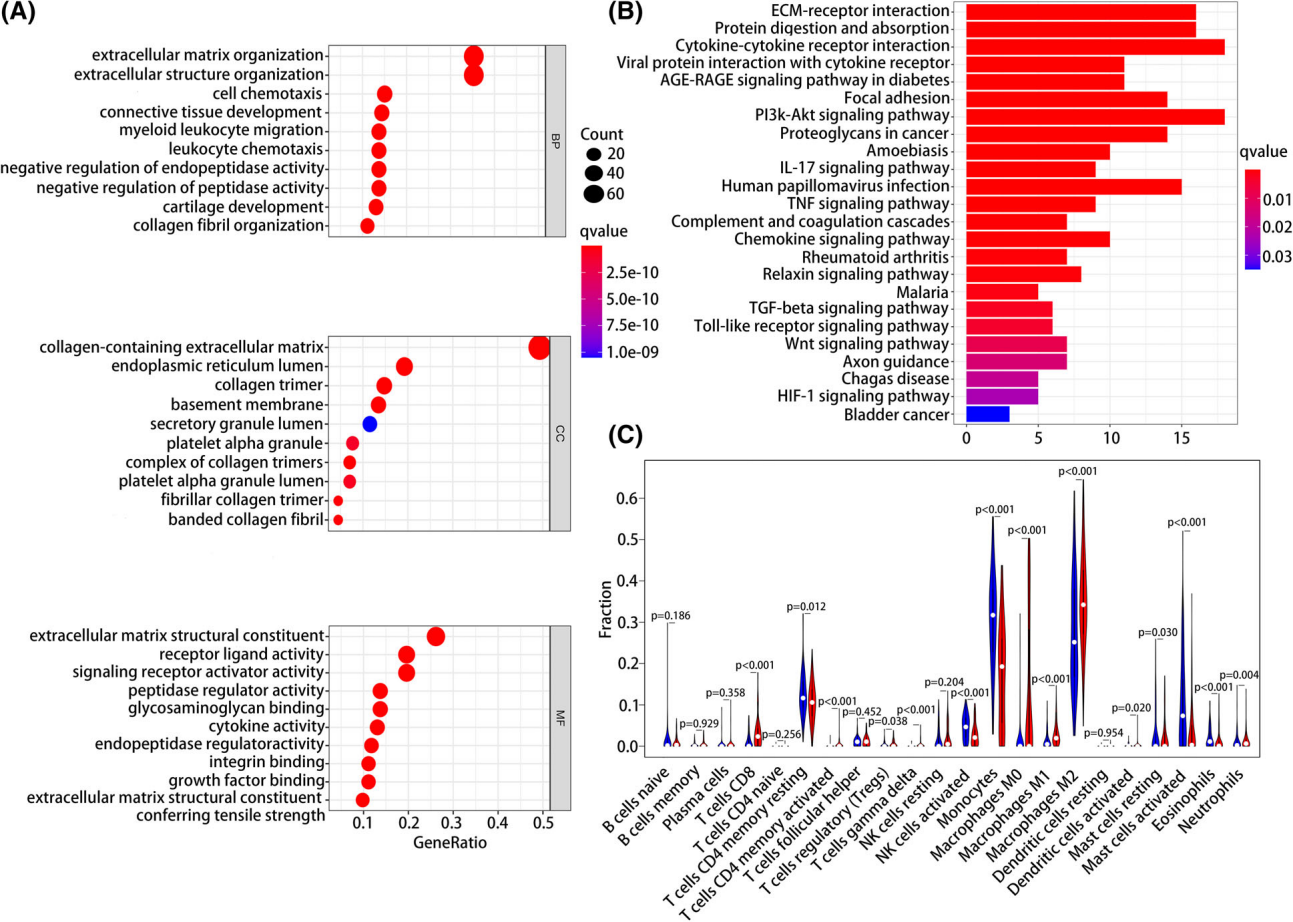
Functional enrichment pathway analysis and immune infiltration of risk signatures. (A) GO analysis. (B) KEGG analysis. (C) Immune cell infiltration in high‐and low‐risk groups.

GSEA of high‐ and low‐risk groups (Fig. 5A) showed that in the GO signaling pathway, biological processes, such as ECM tissue, leukocyte chemotaxis, myeloid leukocyte migration, and cytokine activity, were significantly related to the signature. For the KEGG signaling pathway, immune‐related pathways and proliferation and migration‐related pathways were significantly related to the signature. Immune‐related pathways included viral protein cytokine, cytokine–cytokine receptor interaction, complement and coagulation cascade reaction, tumor necrosis factor signaling pathway, IL17 signaling pathway, toll‐like receptor signaling pathway, chemokine signaling pathway, and transforming growth factor β signaling pathways. Proliferation and migration‐related pathways included the PI3K Akt and Wnt signaling pathways (Fig. 5B).

Given that the high‐ and low‐risk groups were related to immune‐related pathways, we explored the difference in immune cell infiltration between the two groups. There were significant differences in the levels of immune cells including T cells CD8, T cells CD4 memory resting, T cells CD4 memory activated, T cells regulatory (Tregs), T cells gamma delta, monocytes, macrophages M0, macrophages M1, macrophages M2, activated mast cells, activated NK cells, and neutrophils showed significant between the high‐ and low‐risk groups (*P* < 0.05). Analysis of the correlation between risk scores and immune checkpoint expression (Table S5) showed that the number of immune checkpoints, such as CD276, CD274, CTLA4, LAIR1, LILRA5, CD70, and LAG3, was positively related to the risk score. This indicated an immunosuppressive microenvironment in the high‐risk glioma patients.

### Association between tumor mutation burden and treatment response to anti‐PD1/L1 therapy according to the risk signature

**Fig. 6:**
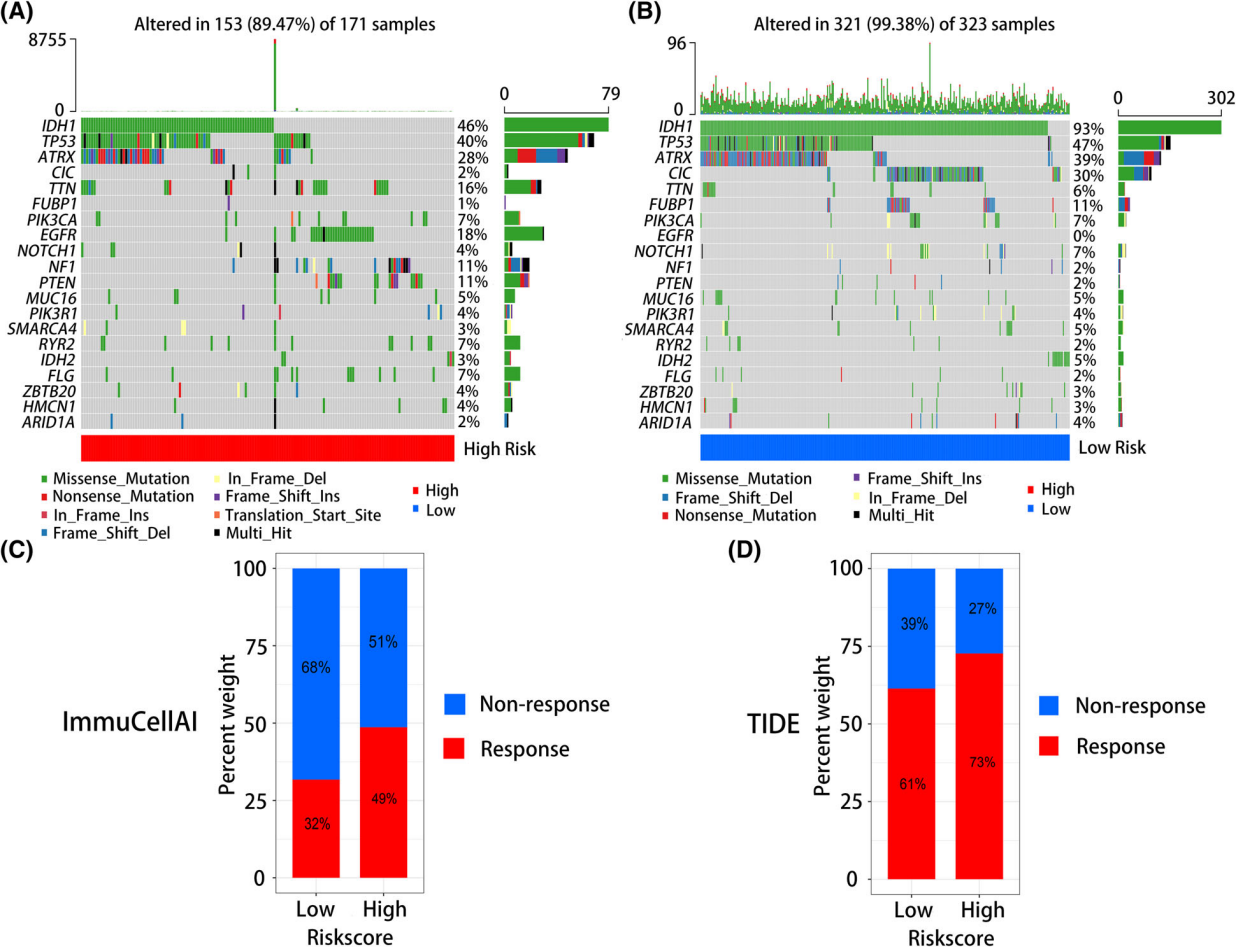
The tumor mutation burdens and anti‐PD1/L1 therapy responses of risk signature. (A) Tumor somatic mutation in high‐risk group. (B) Tumor somatic mutation in low‐risk group. (C) Anti‐ PD1/L1 immunotherapy response of high‐ and low‐risk groups in ImmuCellAI. (D) Anti‐ PD1/L1 immunotherapy response of high‐ and low‐risk groups in TIDE.

As shown in Fig. 6A,B, we selected the top 20 significant mutant genes for analysis. The tumor mutation load was higher in the low‐risk group than in the high‐risk group. Mutant genes accounted for 99.38% and 89.47% of genes in the low‐ and high‐risk groups, respectively. Among them, the mutation frequencies of IDH1 and ATRX mutations were 46% and 28% in the high‐risk group and were 93% and 39% in the low‐risk group, respectively. This indicated that the high‐risk group had worse prognosis. Meanwhile, the mutation frequencies of the oncogenes PTEN and EGFR were higher in the high‐risk group than in the low‐risk group (11% and 18% vs 2% and 0%), indicating more aggressive disease in the high‐risk group.

We use RNA‐seq data in TCGA database to calculate immune infiltration, and score immune infiltration by the expression of each immune cell characteristic gene. Observe the difference of immune cells in different groups and blockade with immune checkpoints, whether there are differences between immune cells in different groups, and the score of each immune cell, and observe whether each sample has a response to immune checkpoint blockade. The results showed that the immune infiltration score of the high‐risk group was higher than that of the low‐risk group, and more patients in the high‐risk group responded to anti‐PD1/PDL1 immunotherapy than in the low‐risk group.

Analysis of the predictive value of immune‐related matrisomes risk signatures for treatment response to anti‐PD1/L1 immunotherapy showed that in TIDE, 73% and 61% of patients in the high‐ and low‐risk groups responded to PD1/L1 immunotherapy, respectively (Fig. 6D). In immuCellAI, 49% and 32% of patients in the high‐ and low‐risk groups responded to PD1/L1 immunotherapy, respectively (Fig. 6C).

### Expression of the eight immune‐related matrisomes

**Fig. 7:**
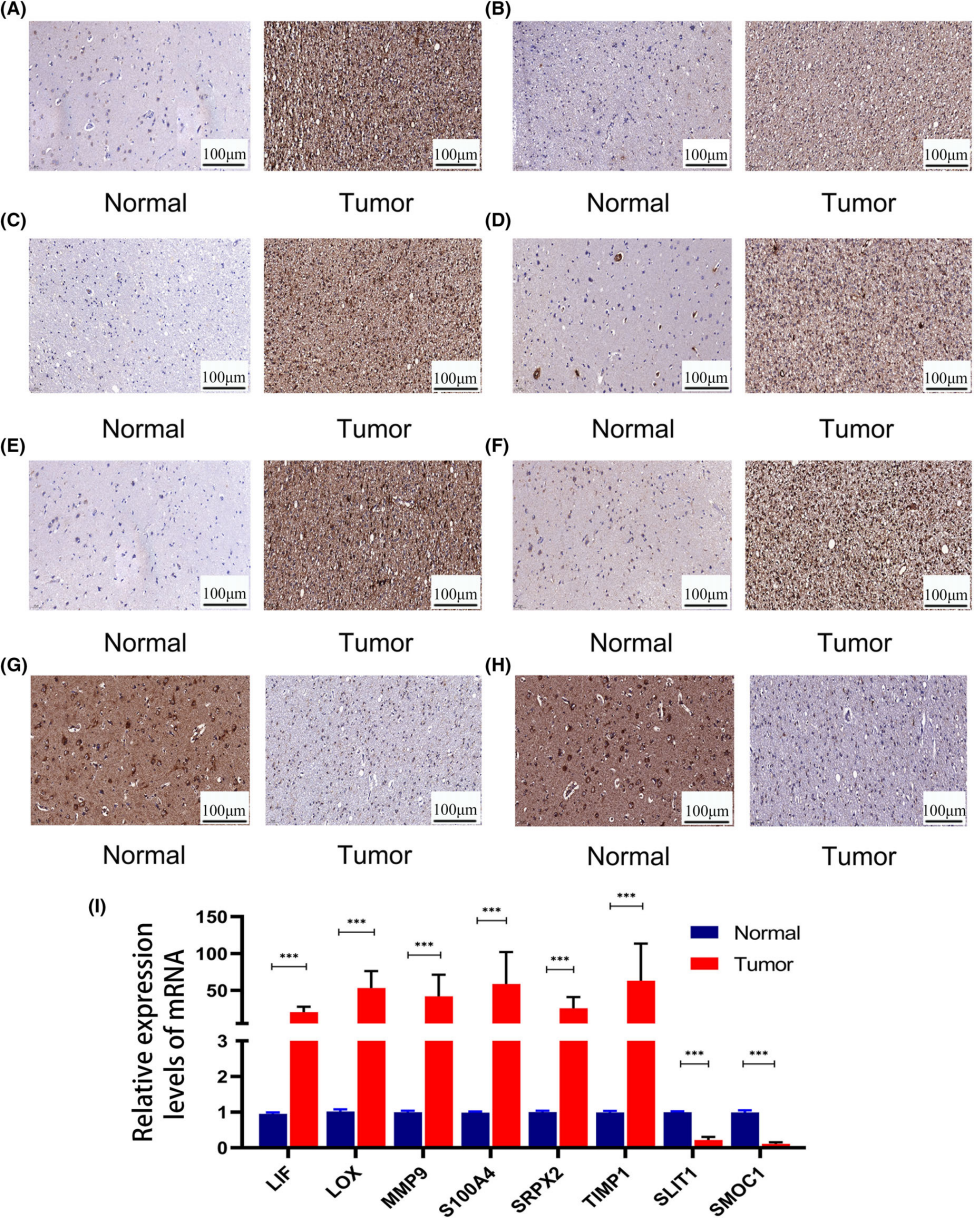
The expression levels of eight immune‐related matrisomes in glioma verified by immunohistochemistry and qPCR. Immunohistochemistry of (A) LIF, (B) LOX, (C) MMP9, (D) S100A4, (E) SRPX2, (F) TIMP1, (G) SLIT1, (H) SMOC1. (I) The mRNA expression levels of eight immune‐related matrisomes (qPCR). Scale bars:100 μm. (I) The mRNA expression levels of eight immune‐related matrisomes were performed in triplicate, and the *t* test was performed. The error bars are presented as the means ± SDs. *P < 0.05, **P < 0.01, and ***P < 0.001.

We verified the mRNA and protein levels of the eight immune‐related matrisomes in brain tissue samples of 26 glioma patients and 18 patients with paracancerous lesions. Quantitative PCR and immunohistochemical analysis showed that LIF, LOX, MMP9, S100A4, SRPX2, and TIMP1 were expressed at high levels in glioma, whereas SLITI1 and SMOC1 were expressed at low levels, consistent with our previous results (Fig. 7; Fig. S4).

## Discussion

Glioma is the most malignant tumor in the brain [25]. Despite several treatment options, including surgery, targeted radiotherapy, and chemotherapy, most patients with GBM die within 2 years [26]. Immunotherapy has made great progress in the treatment of hematological diseases such as lymphoma and leukemia [27, 28]. The main treatment strategies include immune checkpoint inhibitors, monoclonal antibodies, and cell therapy [29]. However, immunotherapy has unsatisfactory benefits for glioma, with patients still having low survival rate possibly owing to the unique immune microenvironment of gliomas [30].

*GBM* is characterized by remarkably unique location, high heterogeneity, and an “immune‐cold” phenotype, promoting an immunosuppressive *microenvironment* [7]. There are only few tumor‐infiltrating lymphocytes (TILs) in the glioma microenvironment, and they often show a state of exhaustion [31, 32]. Further, glioma cells can produce 2,3‐dioxygenase to promote the accumulation of regulatory T cells (Tregs), which can inhibit the function of T cells [33]. Moreover, M2 macrophages secrete proangiogenic factors and immunosuppressive cytokines, which can promote cancer progression. The abundance of immunosuppressive cells in the glioma microenvironment makes it challenging to overcome such immunosuppressive microenvironment [34, 35]. Therefore, it is important to explore the components of the glioma microenvironment.

Extracellular matrix, an important component of the tumor microenvironment, can promote the formation and invasion of tumor [36]. There are many immune cells in the immune microenvironment, including innate immune cells and adaptive immune cells, such as macrophages and lymphocytes. The immune response of TME is mainly related to the composition and activity of infiltrating immune cells, the cell surface expression of immune checkpoint molecules and the changes of related matrix. Tumor‐associated macrophages can secrete different chemokines into tumor tissue. The significant increase of immunosuppressive cells regulated by tumor‐associated macrophages, such as M2 macrophages, regulatory T (Treg) cells, and myeloid‐derived suppressor cells (MDSCs), thus promoting tumor immunosuppression. In addition, some cytokines secreted by activated immune cells, such as interleukin (IL)‐1β, can induce fibroblasts to transform into proinflammatory cells and further promote immunosuppression in TME. Tumor microenvironment (TME) plays a key role in tumor immunosuppression, distant metastasis, local drug resistance and targeted treatment response. The interaction of immune and tumor cells with ECM remodeling may affect tumor metastasis and therapeutic effect [37]. The disorder of ECM is a remarkable feature of cancer. In the process of tumor development, tumor cells will lead to the rigidity of ECM. The signal pathway between cancer cells and ECM activates several important pathways related to mechanical conduction. Considering the increasing importance of the role of matrisomes in tumors and their immune microenvironment, we constructed a risk signature using eight immune‐related matrisomes, namely, LIF, LOX, MMP9, S100A4, SRPX2, SLIT1, SMOC1, and TIMP1, to predict the survival and prognosis of glioma patients.

Tumor cells and tumor associated fibroblasts can secrete leukemia inhibitory factor (LIF) and interleukin‐6 (IL‐6) to promote ECM remodeling and provide conditions for tumor cell migration. It is reported that LIF is related to the adhesion of extravillous trophoblast (EVT) cells and promotes the adhesion of EVT cells. In addition, LIF can promote neural stem cell self‐renewal in the brain [38]. It can also mediate tumor growth factor‐β to induce self‐renewal of glioma‐initiating cells (GICs) and prevent their differentiation [39]. LOX family members can act on the cross‐linking of liver structural ECM and tumor microenvironment. Studies have shown that the expression of LOX family members is up‐regulated in invasive and metastatic tumors, and high expression is associated with poor survival. And they play a crucial role in tumor proliferation, epithelial mesenchymal transition (EMT), invasion, migration, microenvironment formation and immune regulation. LOX is an independent prognostic factor in patients with LGG, and high LOX expression is related to poor OS and response to targeted molecular therapy in patients with LGG [40]. Matrix metallopeptidase 9 (MMP9) is related to angiogenesis in tumors. MMP9 can induce the expansion of myeloid derived suppressor cells (MDSC) and promote tumor immune escape. The increased expression of MMP9 is associated with a variety of high‐grade tumors, metastasis and angiogenesis. MMP9 inhibition reduced angiogenesis and metastasis. Therefore, MMP9 inhibition can reduce tumor invasion to surrounding tissues. Studies have found that diet induced neutralization of granulocyte macrophage colony stimulating factor (GM‐CSF) in obese mice can significantly reduce tumor angiogenesis, immunosuppression, and the progression of metastatic breast cancer. Similarly, MMP9 inhibition reduced tumor angiogenesis and significantly reduced the growth of metastatic tumors. Therefore, the combination of GM‐CSF neutralization and MMP9 inhibition can synergistically reduce angiogenesis and tumor progression. MMP9 is associated with unfavorable prognosis of gliomas and is positively correlated with the grade of primary and recurrent gliomas [41]. The increase of S100A4 level is related to the content of matrix and immune cells. S100A4 mainly exists in the tumor microenvironment and acts as an extracellular factor on breast cancer cells (BCC) to recruit immune cells to the tumor. S100A4 participates in the recruitment of T lymphocytes and the release of cytokines, thereby stimulating the metastasis of breast cancer. S100A4 stimulates basal like BCCs to secrete cytokines and converts monocytes into Tam like cells, thus having tumor supporting functions. S100A4 is a new marker and regulatory factor of glioma stem cells and a molecular chain of mesenchymal transition and stemness of GBM [42]. SRPX2 plays an important role in tumorigenesis and metastasis. SRPX2 is highly expressed in human esophageal squamous cell carcinoma (ESCC). Knockout of SRPX2 can significantly inhibit the proliferation, migration and invasion of ESCC cells and the epithelial mesenchymal transformation (EMT) process in ESCC cells. What's more SRPX2 can promote epithelial‐mesenchymal transformation in GBM and is also related to temozolomide resistance [43]. TIMP metallopeptidase inhibitor‐1 (TIMP‐1) is a member of the matrix metallopeptidase (MMP) inhibitor family. Since MMPs play important roles in ECM remodeling, growth factor availability, TIMPs are involved in processes such as tumor growth and invasion through their regulation of MMPs. Moreover, many evidences have shown that TIMP‐1 is also able to promote tumor cell proliferation and survival, and studies have shown that TIMP‐1 upregulation is associated with a worse outcome in patients with multiple tumors. High levels of TIMP1 are associated with poor prognosis of GBM. In glioma cells, TIMP1 knockdown can delay tumor growth [44, 45]. SPARC‐related modular calcium‐binding protein1 (SMOC1) maybe an interactor of tenascin‐C. Smoc1 can attenuate the migration effect of tenascin‐C on U87 glioma cells.

Kaplan–Meier curves, time‐varying ROC curves, and a nomogram were used to verify the relation between the risk signature and clinical characteristics and prognosis of glioma patients [46]. The functional enrichment pathway showed that the risk signature was related to tumor immunity, proliferation, and migration. Analysis of immune infiltration showed significant differences in the proportion of immune cells between the high‐ and low‐risk groups. Furthermore, some immune checkpoints, such as CD274 and CTLA4, are related to the risk signature. Therefore, it is necessary to establish predictors that can reflect the somatic mutation burdens in the tumor microenvironment and patient prognosis. This will be helpful in understanding the composition of the tumor microenvironment and predicting response to immunotherapy.

We also found that the high‐risk group was more sensitive to anti‐PD1/L1 immunotherapy than is the low‐risk group, possibly due to the higher expression of immune checkpoints in the high‐risk group. The eight immune‐related matrisomes identified may potentially help in the diagnosis and immunotherapy of glioma.

## Conclusions

We screened and verified eight immune‐related matrisomes and established a risk signature. The results showed that the risk signatures were significantly related to the prognosis of glioma patients and immune infiltration of the tumor microenvironment. Thus, they can serve as an indicator to predict the prognosis and immunotherapy response of glioma patients.

## Conflict of interest

The authors declare no conflict of interest.

## Author contributions

XJ and XW were involved in conceptualization. MW was involved in methodology, validation, data curation and writing—review and editing. HY was involved in software, formal analysis, writing—original draft preparation, and visualization. XJ was involved in project administration All authors have read and agreed to the published version of the manuscript.

## Supporting information


**Fig. S1.** The Kaplan Meier (KM) curve showed the overall survival rate of the 8 immune‐related matrisomes. (A) LIF (B) LOX (C) MMP9 (D) S100A4 (E) SRPX2 (F) TIMP1 (G) SLIT1 (H) SMOC1.

**Fig. S2.** Relationship between risk signature and clinical characteristics of glioma in CGGA database. (A) The heatmap showed the relationship between high and low risk groups and clinical features of glioma patients. Risk scores of high and low groups of (B) Age, (C) Gender, (D) IDH mutation status, (E) Grade, (F) Chemotherapy, and (G) Radiotherapy. (H) Univariable Cox regression of 8 immune‐related matrisomes in CGGA database. (B)‐(G) were performed in triplicate, and the t test was performed. *P < 0.05, **P < 0.01, and ***P < 0.001.

**Fig. S3.** Calibration plots were used to validate the efficacy in the CGGA cohort (C‐E). ROC curves were used to evaluate the predictive ability of the nomogram and other predictors (F‐H). Univariate Cox regression analysis (A) and Multivariate Cox regression analysis (B) in CGGA database. All data was performed in triplicate. The error bars are presented as the means ± SDs.

**Fig. S4.** Immunoreactive scores (IRS) of expression of eight immune related matrixes in glioma and adjacent tissues. (A) LIF. (B) LOX. (C) MMP9. (D) S100A4. (E) SRPX2. (F) TIMP1. (G) SLIT1. (H) SMOC1. (A)‐(H) were performed in triplicate. The error bars are presented as the means ± SDs.

**Fig. S5.** Establish and analyze immune‐related matrisomes risk signatures for the prognosis of glioma in CGGA database. (A) The Kaplan Meier (KM) curve showed that the overall survival rate in the high‐risk group was worse than that in the low‐risk group in GEO database under accession number GSE150604. (B) The risk curve and scatter plot of high and low group in GEO database under accession number GSE150604. (C) The AUCs of the 1‐year, 3‐year and 5‐year survival rates in GEO database under accession number GSE150604. (D) Univariate and (E) Multivariate Cox regression analyses were used to verify the prognostic value of risk signature.

**Fig. S6.** Relationship between risk signature and clinical characteristics of glioma in GEO database. (A) The heatmap showed the relationship between high and low risk groups and clinical features of glioma patients. Risk scores of high and low groups of (B) Age, (C) Gender, (D) Grade, (E) Karnofsky Performance Score, (F) MGMT promoter methylation status, and (G) Mini‐mental State Examination (MMSE). (H) Univariable Cox regression of 8 immune‐related matrisomes in GEO database under accession number GSE150604. (B)‐(G) were performed in triplicate, and the t test was performed. *P < 0.05, **P < 0.01, and ***P < 0.001.

**Fig. S7.** Calibration plots were used to validate the efficacy in GEO database under accession number GSE150604 (A‐C). ROC curves were used to evaluate the predictive ability of the nomogram and other predictors (D‐F). All data was performed in triplicate. The error bars are presented as the means ± SDs.

**Fig. S8.** The flow chart of the research route of this paper.

**Fig. S9.** The decision curve analysis of nomogram and other predictors. (A) age. (B) 1p/19q codeletion. (C) Grade. (D) IDH mutation status. (E) Riskscore.

**Fig. S10.** The proportional hazard assumption of the nomogram.

**Table S1.** The comparison of clinicopathological characteristics between the high‐risk and low‐risk groups in the TCGA cohort.
**Table S2.** The comparison of clinicopathological characteristics between the high‐risk and low‐risk groups in the CGGA cohort.
**Table S3.** The expression of tissues used for qRT‐PCR.
**Table S4.** The primers sequence used in this study.
**Table S5.** Correlation between risk score and expression of immune checkpoints.
**Table S6.** The clinical features of 26 GBM patients.
**Table S7.** 119 differentially expressed genes between the low and medium infiltration group.
**Table S8.** 76 differentially expressed between the medium and high infiltration groups.
**Table S9.** 36 matisomes in Lasso analysis.
**Table S10.** The comparison of clinicopathological characteristics between the high‐risk and low‐risk groups in the GEO database.

## Data Availability

All RNA‐seq files are available from the TCGA database (https://cancergenome.nih.gov/) and CGGA (http://www.cgga.org.cn/) database within the article. GEO database under accession number GSE150604.
