# Supplementary material for: Immune‐related matrisomes are potential biomarkers to predict the prognosis and immune microenvironment of glioma patients
Source: FEBS Open Bio. 2022 Dec 30;13(2):307–22. doi: 10.1002/2211-5463.13541 (PMC9900094; doi:10.1002/2211-5463.13541)
Supplement: Supplementary file 1 — Fig. S1. The Kaplan Meier (KM) curve showed the overall survival rate of the 8 immune‐related matrisomes. (A) LIF (B) LOX (C) MMP9 (D) S100A4 (E) SRPX2 (F) TIMP1 (G) SLIT1 (H) SMOC1. [file FEB4-13-307-s003.docx]

**
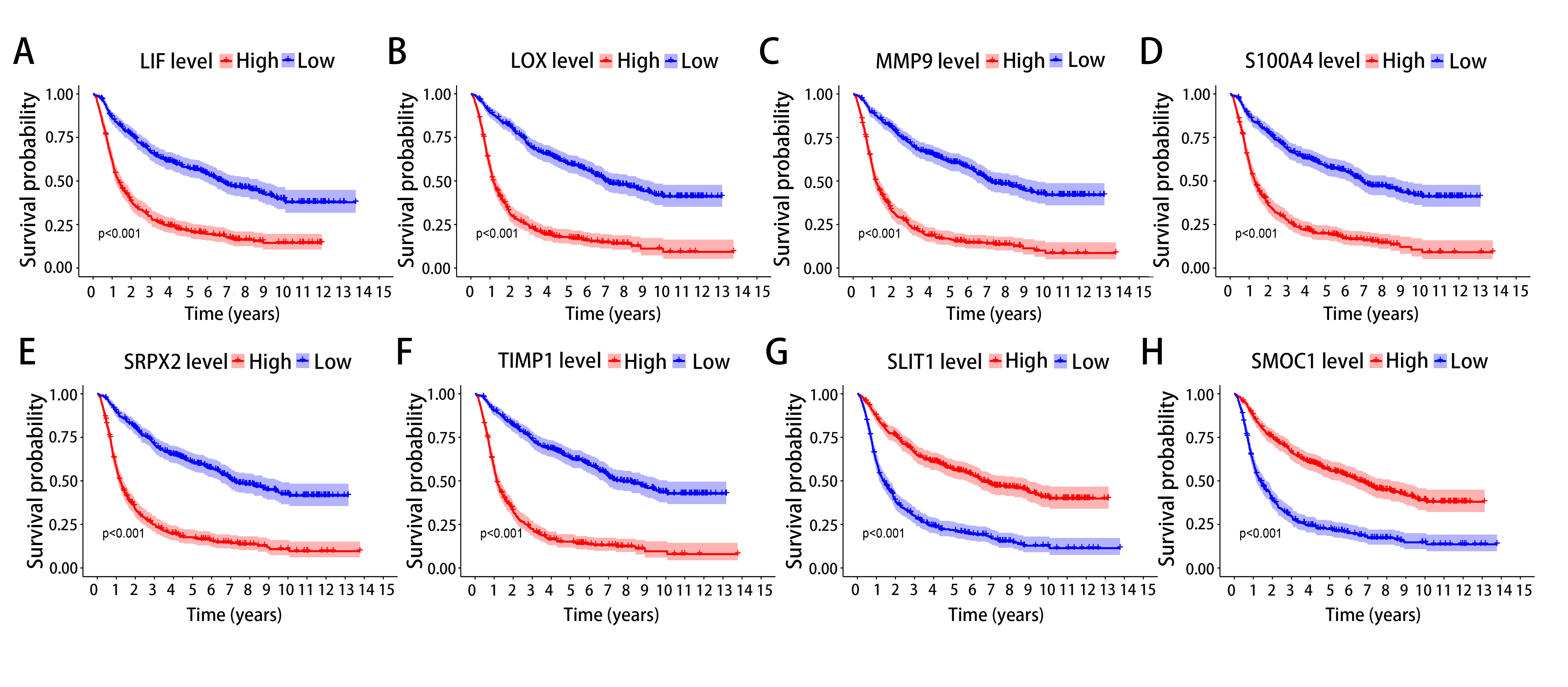
Supplementary Figure S1. The Kaplan Meier (KM) curve showed the overall survival rate of the 8 immune-related matrisomes.** (A) LIF (B) LOX (C) MMP9 (D) S100A4 (E) SRPX2 (F) TIMP1 (G) SLIT1 (H) SMOC1.
