# Supplementary material for: Immune‐related matrisomes are potential biomarkers to predict the prognosis and immune microenvironment of glioma patients
Source: FEBS Open Bio. 2022 Dec 30;13(2):307–22. doi: 10.1002/2211-5463.13541 (PMC9900094; doi:10.1002/2211-5463.13541)
Supplement: Supplementary file 2 — Fig. S2. Relationship between risk signature and clinical characteristics of glioma in CGGA database. (A) The heatmap showed the relationship between high and low risk groups and clinical features of glioma patients. Risk scores of high and low groups of (B) Age, (C) Gender, (D) IDH mutation status, (E) Grade, (F) Chemotherapy, and (G) Radiotherapy. (H) Univariable Cox regression of 8 immune‐related matrisomes in CGGA database. (B)‐(G) were performed in triplicate, and the t test was performed. *P < 0.05, **P < 0.01, and ***P < 0.001. [file FEB4-13-307-s010.docx]

**
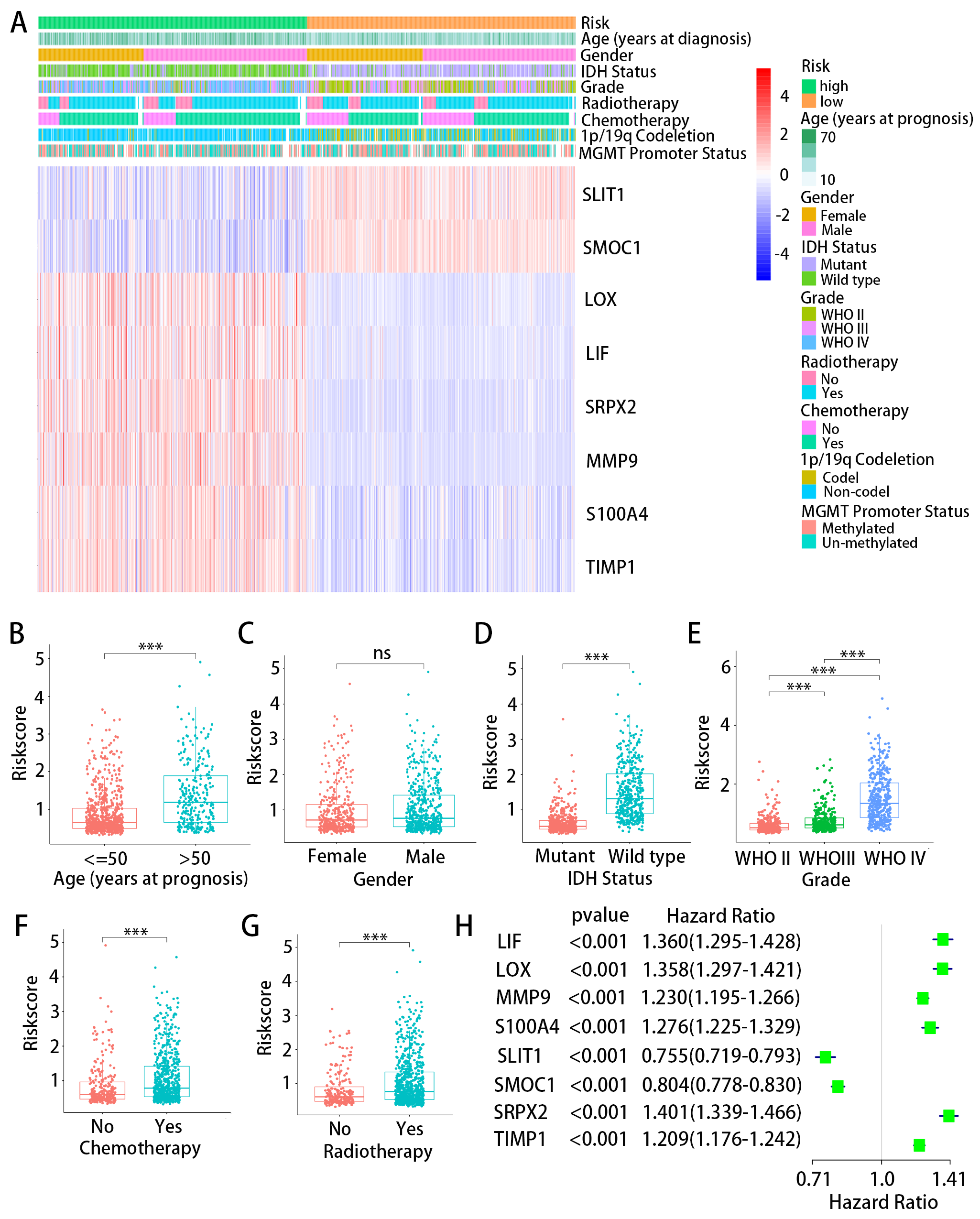
Supplementary Figure S2. Relationship between risk signature and clinical characteristics of glioma in CGGA database.** (A) The heatmap showed the relationship between high and low risk groups and clinical features of glioma patients. Risk scores of high and low groups of (B) Age, (C) Gender, (D) IDH mutation status, (E) Grade, (F) Chemotherapy, and (G) Radiotherapy. (H) Univariable Cox regression of 8 immune-related matrisomes in CGGA database. (B)-(G) were performed in triplicate, and the t test was performed. *P < 0.05, **P < 0.01, and ***P < 0.001.
