# Supplementary material for: Immune‐related matrisomes are potential biomarkers to predict the prognosis and immune microenvironment of glioma patients
Source: FEBS Open Bio. 2022 Dec 30;13(2):307–22. doi: 10.1002/2211-5463.13541 (PMC9900094; doi:10.1002/2211-5463.13541)
Supplement: Supplementary file 3 — Fig. S3. Calibration plots were used to validate the efficacy in the CGGA cohort (C‐E). ROC curves were used to evaluate the predictive ability of the nomogram and other predictors (F‐H). Univariate Cox regression analysis (A) and Multivariate Cox regression analysis (B) in CGGA database. All data was performed in triplicate. The error bars are presented as the means ± SDs. [file FEB4-13-307-s005.docx]

**
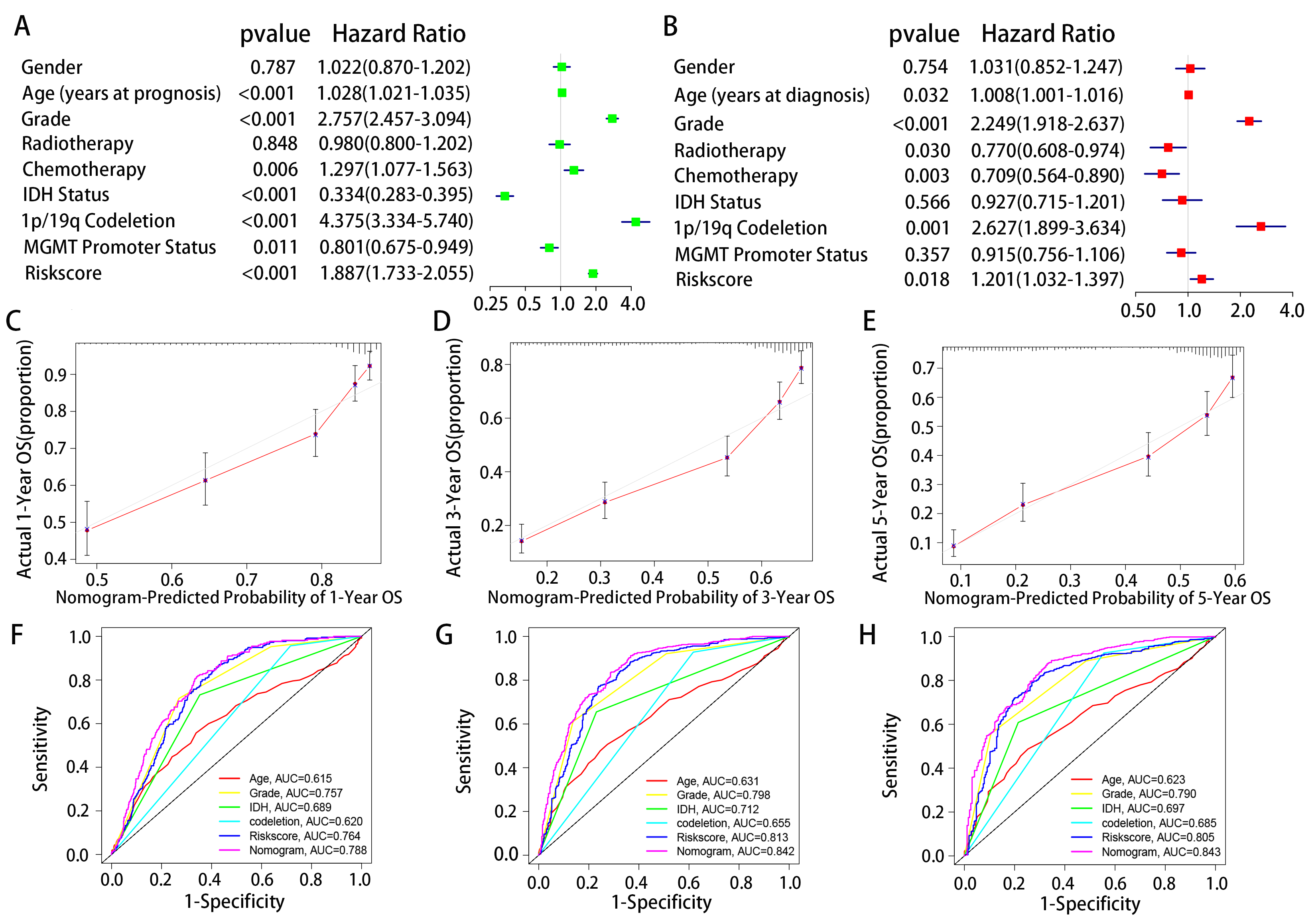
Supplementary Figure S3.** Calibration plots were used to validate the efficacy in the CGGA cohort (C-E). ROC curves were used to evaluate the predictive ability of the nomogram and other predictors (F-H). Univariate Cox regression analysis (A) and Multivariate Cox regression analysis (B) in CGGA database. All data was performed in triplicate. The error bars are presented as the means ± SDs.
