# Supplementary material for: Immune‐related matrisomes are potential biomarkers to predict the prognosis and immune microenvironment of glioma patients
Source: FEBS Open Bio. 2022 Dec 30;13(2):307–22. doi: 10.1002/2211-5463.13541 (PMC9900094; doi:10.1002/2211-5463.13541)
Supplement: Supplementary file 4 — Fig. S4. Immunoreactive scores (IRS) of expression of eight immune related matrixes in glioma and adjacent tissues. (A) LIF. (B) LOX. (C) MMP9. (D) S100A4. (E) SRPX2. (F) TIMP1. (G) SLIT1. (H) SMOC1. (A)‐(H) were performed in triplicate. The error bars are presented as the means ± SDs. [file FEB4-13-307-s011.docx]

**
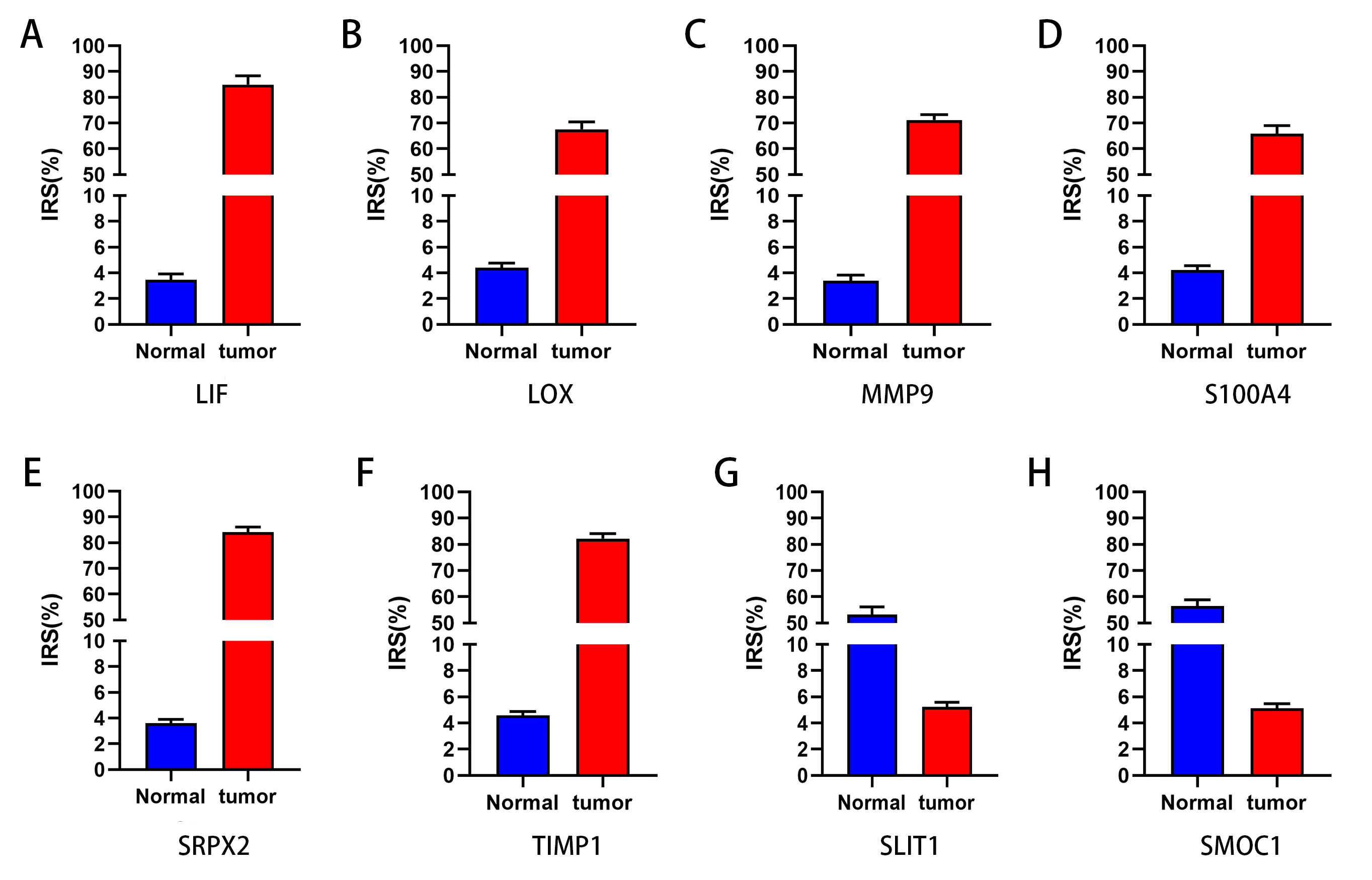
Supplementary Figure S4.** Immunoreactive scores (IRS) of expression of eight immune related matrixes in glioma and adjacent tissues. (A) LIF. (B) LOX. (C) MMP9. (D) S100A4. (E) SRPX2. (F) TIMP1. (G) SLIT1. (H) SMOC1. (A)-(H) were performed in triplicate. The error bars are presented as the means ± SDs.
