# Supplementary material for: Immune‐related matrisomes are potential biomarkers to predict the prognosis and immune microenvironment of glioma patients
Source: FEBS Open Bio. 2022 Dec 30;13(2):307–22. doi: 10.1002/2211-5463.13541 (PMC9900094; doi:10.1002/2211-5463.13541)
Supplement: Supplementary file 5 — Fig. S5. Establish and analyze immune‐related matrisomes risk signatures for the prognosis of glioma in CGGA database. (A) The Kaplan Meier (KM) curve showed that the overall survival rate in the high‐risk group was worse than that in the low‐risk group in GEO database under accession number GSE150604. (B) The risk curve and scatter plot of high and low group in GEO database under accession number GSE150604. (C) The AUCs of the 1‐year, 3‐year and 5‐year survival rates in GEO database under accession number GSE150604. (D) Univariate and (E) Multivariate Cox regression analyses were used to verify the prognostic value of risk signature. [file FEB4-13-307-s008.docx]

**
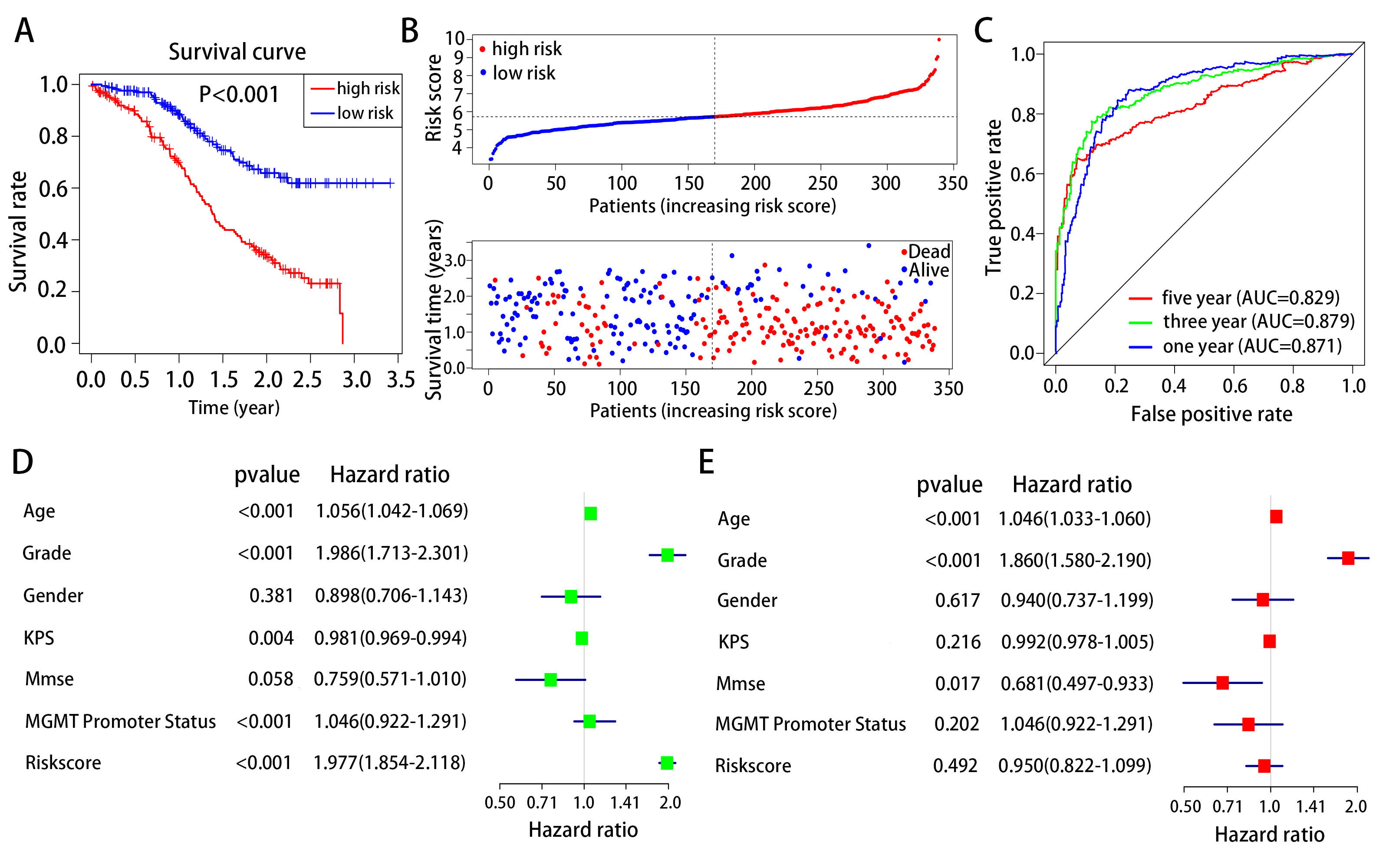
Supplementary Figure S5. Establish and analyze immune-related matrisomes risk signatures for the prognosis of glioma in CGGA database.** (A) The Kaplan Meier (KM) curve showed that the overall survival rate in the high-risk group was worse than that in the low-risk group in GEO database under accession number GSE150604. (B) The risk curve and scatter plot of high and low group in GEO database under accession number GSE150604. (C) The AUCs of the 1-year, 3-year and 5-year survival rates in GEO database under accession number GSE150604. (D) Univariate and (E) Multivariate Cox regression analyses were used to verify the prognostic value of risk signature.
