# Supplementary material for: Immune‐related matrisomes are potential biomarkers to predict the prognosis and immune microenvironment of glioma patients
Source: FEBS Open Bio. 2022 Dec 30;13(2):307–22. doi: 10.1002/2211-5463.13541 (PMC9900094; doi:10.1002/2211-5463.13541)
Supplement: Supplementary file 6 — Fig. S6. Relationship between risk signature and clinical characteristics of glioma in GEO database. (A) The heatmap showed the relationship between high and low risk groups and clinical features of glioma patients. Risk scores of high and low groups of (B) Age, (C) Gender, (D) Grade, (E) Karnofsky Performance Score, (F) MGMT promoter methylation status, and (G) Mini‐mental State Examination (MMSE). (H) Univariable Cox regression of 8 immune‐related matrisomes in GEO database under accession number GSE150604. (B)‐(G) were performed in triplicate, and the t test was performed. *P < 0.05, **P < 0.01, and ***P < 0.001. [file FEB4-13-307-s009.docx]

**
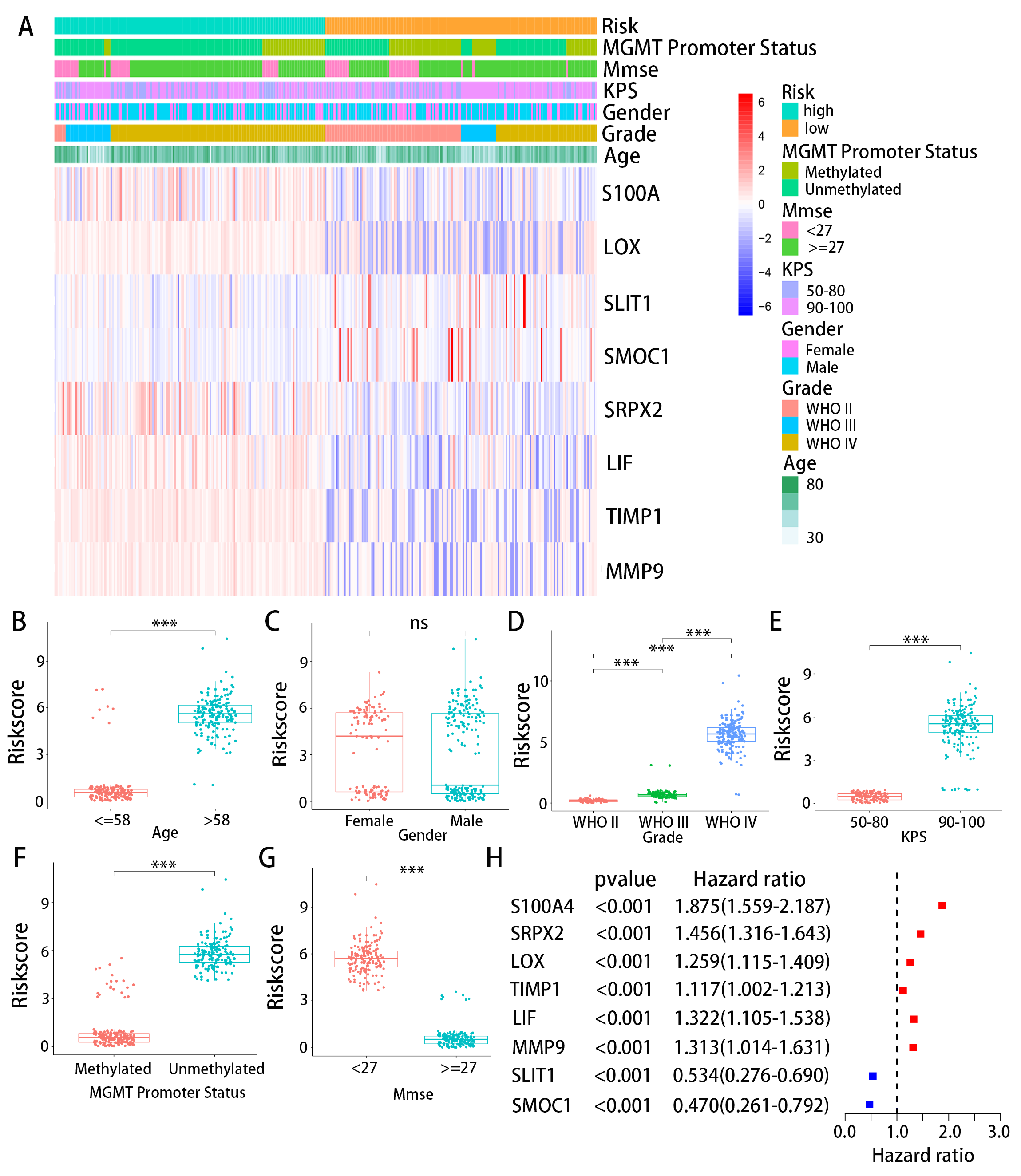
Supplementary Figure S6. Relationship between risk signature and clinical characteristics of glioma in GEO database.** (A) The heatmap showed the relationship between high and low risk groups and clinical features of glioma patients. Risk scores of high and low groups of (B) Age, (C) Gender, (D) Grade, (E) Karnofsky Performance Score, (F) MGMT promoter methylation status, and (G) Mini-mental State Examination (MMSE). (H) Univariable Cox regression of 8 immune-related matrisomes in GEO database under accession number GSE150604. (B)-(G) were performed in triplicate, and the t test was performed. *P < 0.05, **P < 0.01, and ***P < 0.001.
