# Supplementary material for: Immune‐related matrisomes are potential biomarkers to predict the prognosis and immune microenvironment of glioma patients
Source: FEBS Open Bio. 2022 Dec 30;13(2):307–22. doi: 10.1002/2211-5463.13541 (PMC9900094; doi:10.1002/2211-5463.13541)
Supplement: Supplementary file 7 — Fig. S7. Calibration plots were used to validate the efficacy in GEO database under accession number GSE150604 (A‐C). ROC curves were used to evaluate the predictive ability of the nomogram and other predictors (D‐F). All data was performed in triplicate. The error bars are presented as the means ± SDs. [file FEB4-13-307-s001.docx]

**
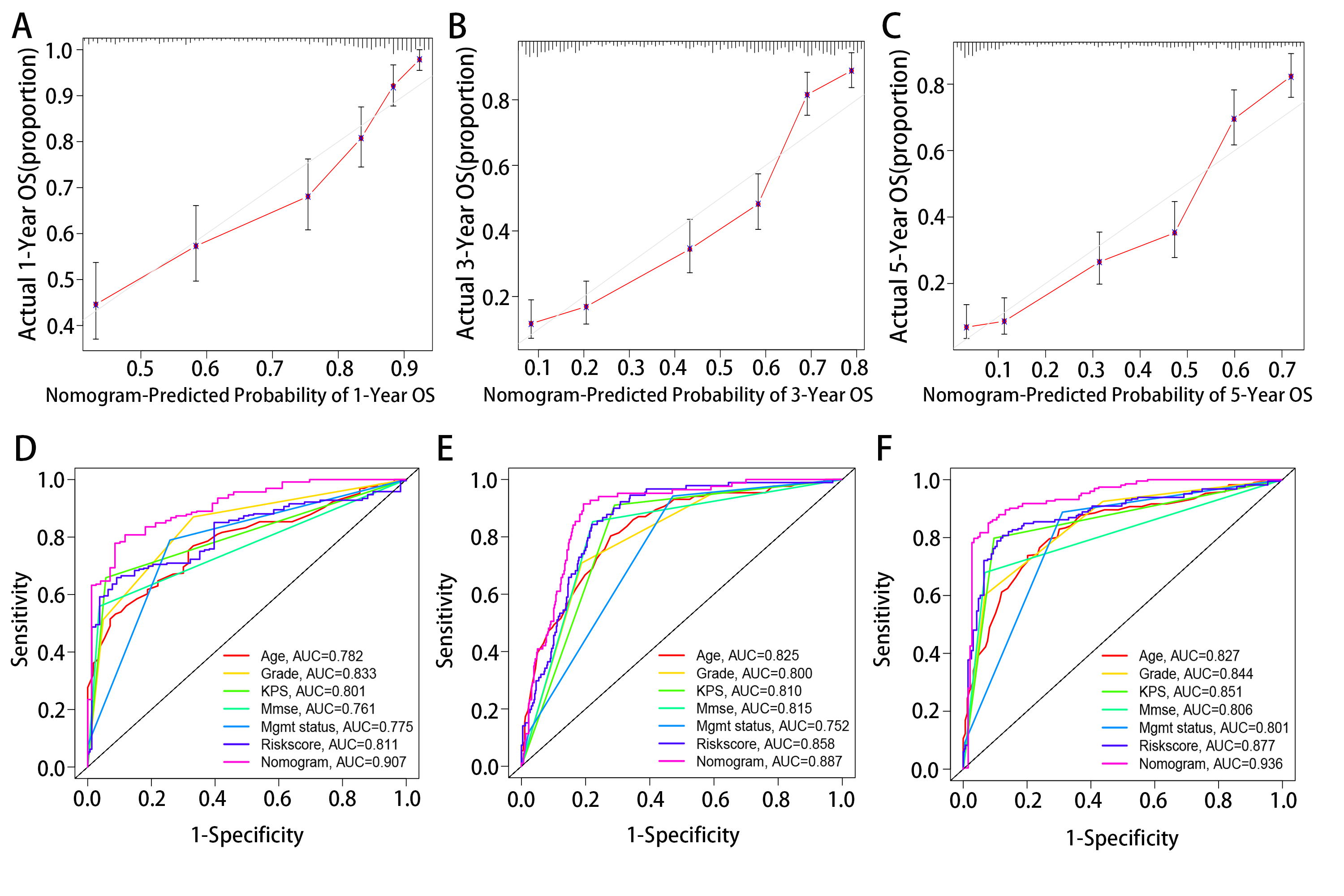
Supplementary Figure** **S7.** Calibration plots were used to validate the efficacy in GEO database under accession number GSE150604 (A-C). ROC curves were used to evaluate the predictive ability of the nomogram and other predictors (D-F). All data was performed in triplicate. The error bars are presented as the means ± SDs.
