# Supplementary figures and images for: Immune‐related matrisomes are potential biomarkers to predict the prognosis and immune microenvironment of glioma patients
Source: FEBS Open Bio. 2022 Dec 30;13(2):307–22. doi: 10.1002/2211-5463.13541 (PMC9900094; doi:10.1002/2211-5463.13541)

**
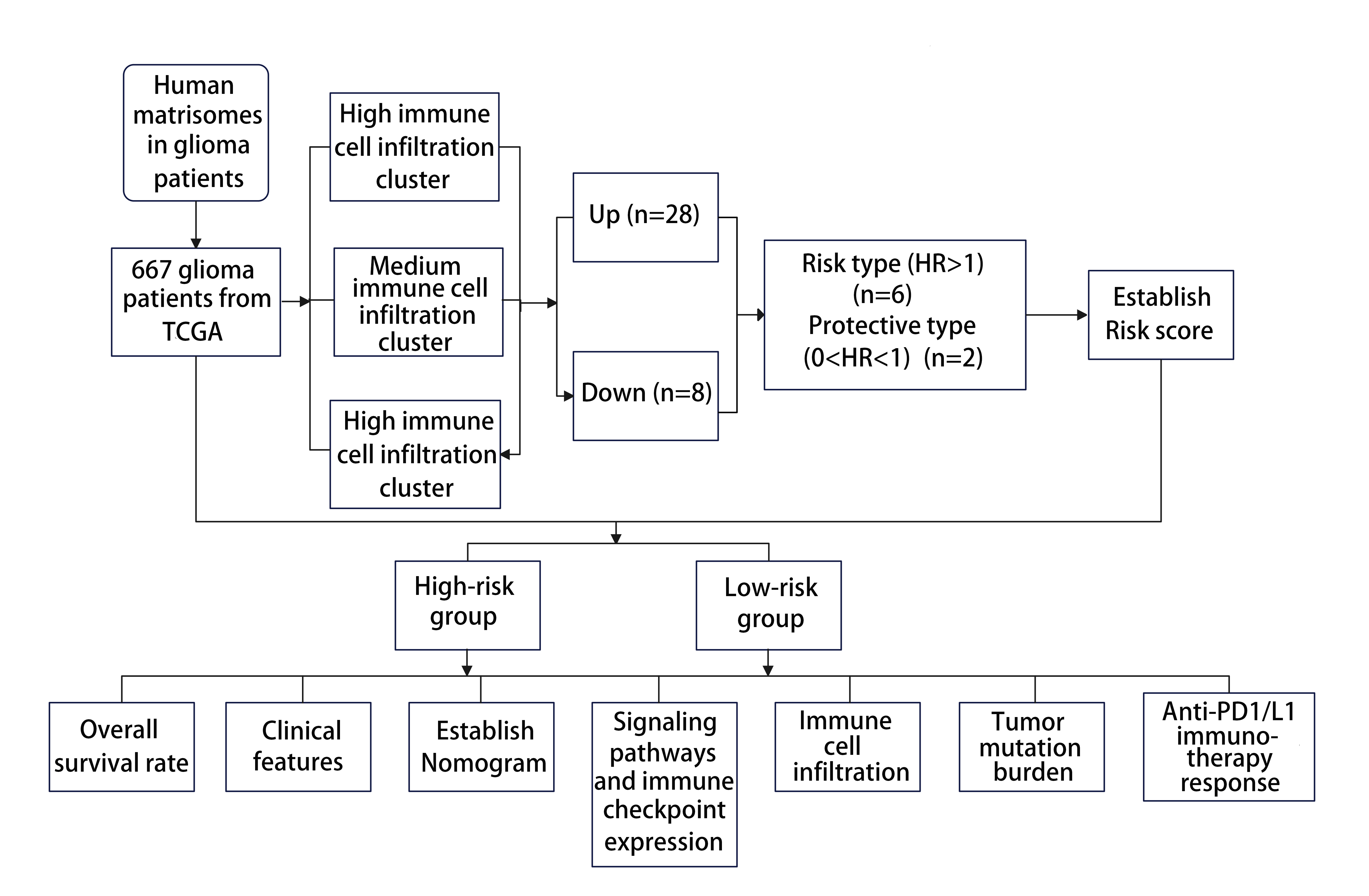
Supplementary Figure S8.** The flow chart of the research route of this paper.

Supplement: Supplementary file 8 — Fig. S8. The flow chart of the research route of this paper. [file FEB4-13-307-s006.docx]

**
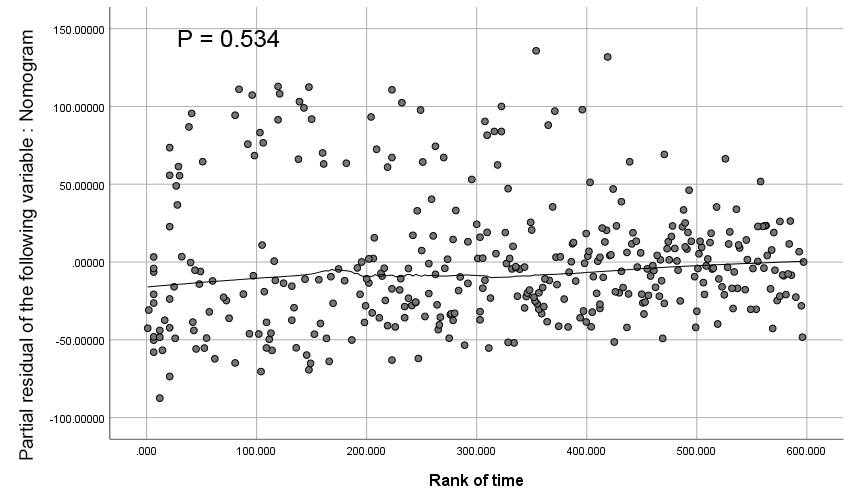
Supplementary Figure S10.** Theproportional hazard assumption of the nomogram.

Supplement: Supplementary file 10 — Fig. S10. The proportional hazard assumption of the nomogram. [file FEB4-13-307-s007.docx]
