# Supplementary material for: Immune‐related matrisomes are potential biomarkers to predict the prognosis and immune microenvironment of glioma patients
Source: FEBS Open Bio. 2022 Dec 30;13(2):307–22. doi: 10.1002/2211-5463.13541 (PMC9900094; doi:10.1002/2211-5463.13541)
Supplement: Supplementary file 9 — Fig. S9. The decision curve analysis of nomogram and other predictors. (A) age. (B) 1p/19q codeletion. (C) Grade. (D) IDH mutation status. (E) Riskscore. [file FEB4-13-307-s002.docx]

**
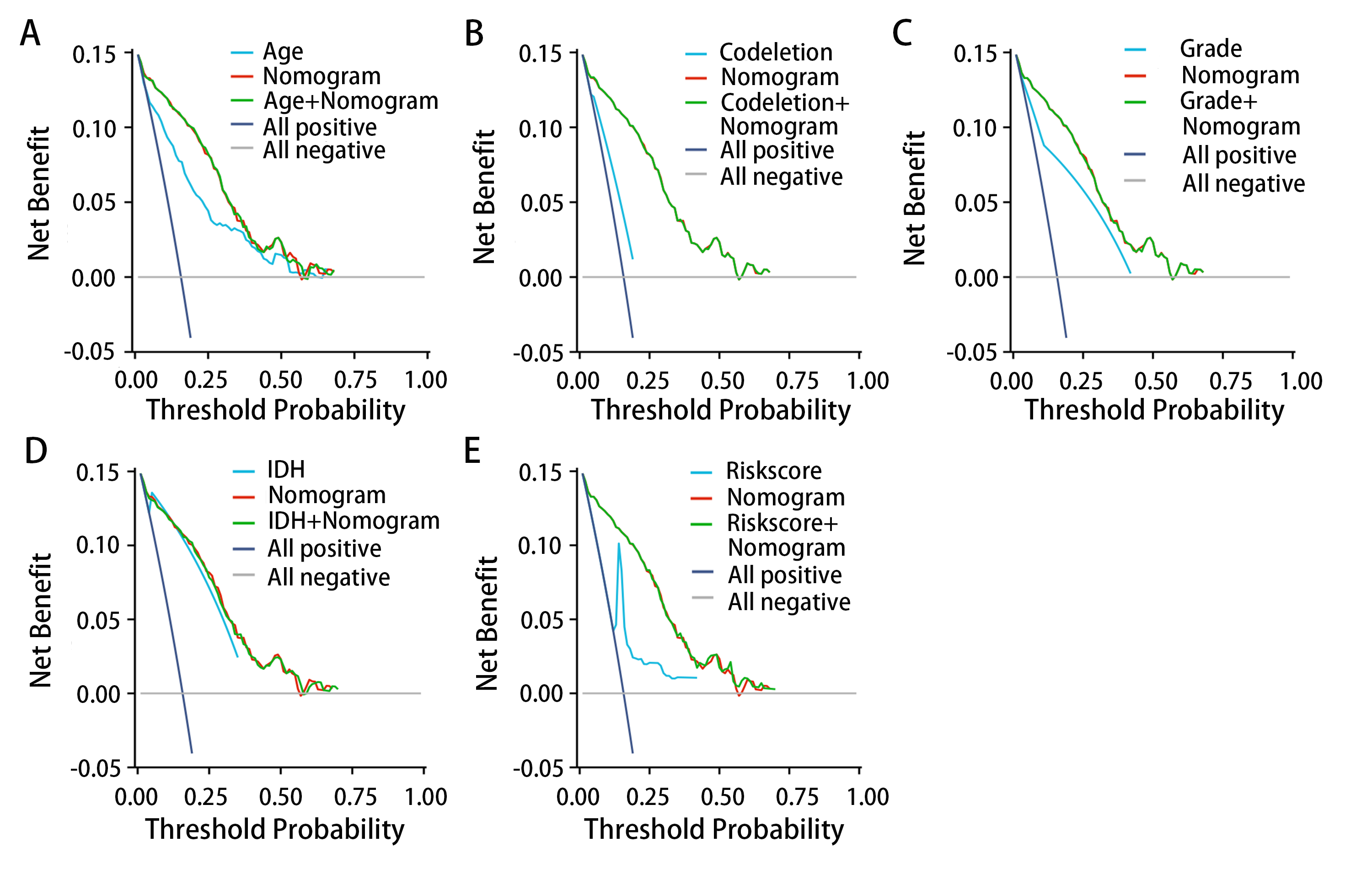
Supplementary Figure S9.** The decision curve analysis of nomogram and other predictors. (A) age. (B) 1p / 19q codeletion. (C) Grade. (D) IDH mutation status. (E) Riskscore.
