# Supplementary material for: Immune‐related matrisomes are potential biomarkers to predict the prognosis and immune microenvironment of glioma patients
Source: FEBS Open Bio. 2022 Dec 30;13(2):307–22. doi: 10.1002/2211-5463.13541 (PMC9900094; doi:10.1002/2211-5463.13541)
Supplement: Supplementary file 11 — Table S1. The comparison of clinicopathological characteristics between the high‐risk and low‐risk groups in the TCGA cohort. Table S2. The comparison of clinicopathological characteristics between the high‐risk and low‐risk groups in the CGGA cohort. Table S3. The expression of tissues used for qRT‐PCR. Table S4. The primers sequence used in this study. Table S5. Correlation between risk score and expression of immune checkpoints. Table S6. The clinical features of 26 GBM patients. Table S7. 119 differentially expressed genes between the low and medium infiltration group. Table S8. 76 differentially expressed between the medium and high infiltration groups. Table S9. 36 matisomes in Lasso analysis. Table S10. The comparison of clinicopathological characteristics between the high‐risk and low‐risk groups in the GEO database. [file FEB4-13-307-s004.docx]

**Supplementary Table S1. The comparison of clinicopathological characteristics between the high-risk and low-risk groups in the TCGA cohort**

| **Clinicopathological characteristics** | **High-risk group**  **(n = 315)** | **Low-risk group**  **(n = 352)** | ***p-*value** |
| --- | --- | --- | --- |
| **Age** (Mean ± SD) | 45.26 ± 14.62 | 48.43 ± 15.48 | <0.001 |
| **Gender** |  |  | 0.231 |
| Male | 182 (60.5%) | 174 (56.1%) |  |
| Female | 119 (39.5%) | 136 (43.9%) |  |
| **WHO grade** |  |  | <0.001 |
| II | 59 (19.6%) | 156 (50.3%) | <0.001 |
| III | 103 (34.2%) | 133 (42.9%) |  |
| IV | 139 (46.2%) | 21 (6.8%) |  |
| **IDH status** |  |  |  |
| Mutant | 127 (41.2%) | 274 (78.3%) |  |
| Wild type | 181 (58.8%) | 76 (21.7%) |  |
| **1p/19q codeletion** |  |  | <0.001 |
| Codel | 18 (5.8%) | 148 (42.3%) |  |
| Non-codel | 293 (94.2%) | 202 (57.7%) | <0.001 |
| **MGMT promoter status** |  |  | <0.001 |
| Methylated | 251(83.1%) | 221 (66.8%) |  |
| Unmethylated | 51 (16.9%) | 110 (33.2%) |  |
|  |  |  |  |
|  |  |  |  |
|  |  |  |  |
|  |  |  |  |
|  |  |  |  |
|  |  |  |  |
|  |  |  |  |
|  |  |  |  |
|  |  |  |  |

**Supplementary Table S2. The comparison of clinicopathological characteristics between the high-risk and low-risk groups in the CGGA cohort**

| **Clinicopathological characteristics** | **High-risk group**  **(n = 485)** | **Low-risk group**  **(n = 485)** | ***p-*value** |
| --- | --- | --- | --- |
| **Age** (Mean ± SD) | 46.39 ± 13.45 | 40.33 ± 10.11 | <0.001 |
| **Gender** |  |  | 0.286 |
| Male | 295 (60.8%) | 276 (56.9%) |  |
| Female | 190 (39.2%) | 209 (43.1%) |  |
| **WHO grade** |  |  | <0.001 |
| II | 55 (11.4%) | 215 (44.3%) | <0.001 |
| III | 114 (23.7%) | 208 (42.9%) |  |
| IV | 312 (64.9%) | 62 (12.8%) |  |
| **IDH status** |  |  |  |
| Mutant | 108 (22.8%) | 392 (87.5%) |  |
| Wild type | 365 (77.2%) | 56 (12.5%) |  |
| **1p/19q codeletion** |  |  | <0.001 |
| Codel | 28 (6.3%) | 171 (38.0%) |  |
| Non-codel | 418 (93.7%) | 279 (62.0%) |  |
| **MGMT promoter status** |  |  | <0.001 |
| Methylated | 221 (53.6%) | 235 (58.0%) |  |
| Unmethylated | 191 (46.4%) | 170 (42.0%) |  |
|  |  |  |  |
|  |  |  |  |
|  |  |  |  |
|  |  |  |  |
|  |  |  |  |
|  |  |  |  |
|  |  |  |  |
|  |  |  |  |
|  |  |  |  |

**Supplementary Table S3. The expression of tissues used for qRT-PCR.**

| **Gene** | **paracancerous brain tissue** | **Glioma tissues** | **Total** |
| --- | --- | --- | --- |
| LIF | 4 | 10 | 14 |
| LOX | 5 | 8 | 13 |
| MMP9 | 4 | 9 | 13 |
| S100A4 | 4 | 7 | 11 |
| SRPX2 | 6 | 9 | 15 |
| TIMP1 | 5 | 8 | 13 |
| SLIT1 | 4 | 5 | 9 |
| SMOC1 | 4 | 5 | 9 |

**Supplementary Table S4. The Primers sequence used in this study**

| **Name** | **Forward-primer** | **Reverse-primer** |
| --- | --- | --- |
| LIF | 5’-CAAGCGCCGTATGGGACTTT-3’ | 5’-GGAGGCATCCATGTAGCTCT-3’ |
| LOX | 5’-GATACAGCGAGCTGGTGAATG-3’ | 5’-CATCCTCATCGTGCGTACAGT-3’ |
| MMP9 | 5’-TGTACCGCTATGGTTACACTCG-3’ | 5’-GGCAGGGACAGTTGCTTCT-3’ |
| S100A4 | 5’-GATGAGCAACTTGGACAGCAA-3’ | 5’-CTGGGCTGCTTATCTGGGAAG-3’ |
| SRPX2 | 5’-CCACATGCTACTCACCGAAGG-3’ | 5’-GTAGTGCGTGGCATCTCATCT-3’ |
| TIMP1 | 5’-CTTCTGCAATTCCGACCTCGT-3’ | 5’-ACGCTGGTATAAGGTGGTCTG-3’ |
| SLIT1 | 5’-GCCTGGAACTCAATGGCAAC-3’ | 5’-CTGGTTTCGGTTCAGTCGCA-3’ |
| SMOC1 | 5’-AGGTCCTACGAGTCCATGTGT-3’ | 5’-CACTGCACCTGGGTAAAGG-3’ |
| GAPDH | 5’-AAAAGCATCACCCGGAGGAGAA-3’ | 5’-AAGGAAATGAATGGGCAGCCG-3’ |

**Supplementary Table S5. Correlation between risk score and expression of immune checkpoints.**

| **symbol** | **correlation** | **pvalue** |  | **symbol** | **correlation** | **pvalue** | **symbol** | **correlation** | **pvalue** |
| --- | --- | --- | --- | --- | --- | --- | --- | --- | --- |
| CD276 | 0.524520443 | 2.03E-48 |  | TNFRSF10C | 0.357029422 | 1.75E-21 | LILRB1 | 0.34495892 | 4.50E-20 |
| LAIR1 | 0.469684976 | 6.70E-38 |  | CD70 | 0.374751711 | 1.15E-23 | TIGIT | 0.034118547 | 0.378989 |
| CD28 | 0.37841712 | 3.90E-24 |  | CD27 | 0.27027161 | 1.25E-12 | LTBR | 0.43166896 | 1.19E-31 |
| TNFRSF1A | 0.471595162 | 3.09E-38 |  | TNFSF9 | 0.216557371 | 1.61E-08 | CD274 | 0.492080254 | 5.74E-42 |
| TNFRSF25 | 0.078789324 | 0.041933 |  | LILRB4 | 0.239153987 | 3.95E-10 | TMIGD2 | 0.012723983 | 0.742903 |
| NCR3LG1 | -0.20956895 | 4.68E-08 |  | FASLG | 0.284695713 | 6.66E-14 | NCR3 | 0.029860969 | 0.441345 |
| LAIR2 | 0.067802494 | 0.080149 |  | TNFRSF12A | 0.476375787 | 4.39E-39 | EDA | 0.057777602 | 0.136058 |
| BTLA | 0.105599987 | 0.006338 |  | TNFSF10 | 0.513269383 | 4.21E-46 | PVRIG | 0.216573003 | 1.60E-08 |
| VSIG8 | 0.039899063 | 0.303515 |  | LILRB2 | 0.523714205 | 2.99E-48 | LGALS9 | 0.242126778 | 2.36E-10 |
| LILRA5 | 0.513210484 | 4.32E-46 |  | LAG3 | 0.052378186 | 0.176655 | HHLA2 | 0.166553545 | 1.53E-05 |
| TNFRSF14 | 0.421970313 | 3.52E-30 |  | TNFRSF8 | 0.10943253 | 0.004663 | SLAMF6 | 0.380152231 | 2.33E-24 |
| SIRPG | 0.370494303 | 3.95E-23 |  | TNFRSF1B | 0.406925631 | 5.45E-28 | TNFRSF10D | 0.406808803 | 5.66E-28 |
| BTN1A1 | 0.007298358 | 0.850768 |  | RELT | 0.13085545 | 0.000705 | BTN3A1 | 0.134900201 | 0.000477 |
| PVR | 0.39250183 | 5.42E-26 |  | LILRB5 | 0.241646509 | 2.56E-10 | CD47 | 0.184556963 | 1.60E-06 |
| TNFRSF10B | 0.398395089 | 8.50E-27 |  | EDA2R | 0.085883552 | 0.026555 | CTLA4 | 0.192184575 | 5.71E-07 |
| LILRB3 | 0.490627488 | 1.08E-41 |  | TNF | 0.046294518 | 0.232472 | CD160 | 0.019437389 | 0.616299 |
| TNFSF13B | 0.128914372 | 0.000847 |  | TNFRSF17 | 0.09017533 | 0.019844 | SLAMF1 | 0.333523643 | 8.58E-19 |
| TNFRSF11A | 0.400439886 | 4.43E-27 |  | CD86 | 0.386079069 | 3.91E-25 | LILRA2 | 0.2548172 | 2.40E-11 |
| TNFRSF11B | 0.334392549 | 6.89E-19 |  | CD244 | 0.276220454 | 3.81E-13 | TNFRSF13C | 0.193341918 | 4.87E-07 |
| ICOS | 0.343841075 | 6.03E-20 |  | TNFSF14 | 0.253073389 | 3.31E-11 | LTA | 0.011027999 | 0.77619 |
| CD96 | 0.39971868 | 5.58E-27 |  | NECTIN2 | 0.486782602 | 5.60E-41 | TNFSF8 | 0.404901336 | 1.05E-27 |
| LTB | 0.230648805 | 1.67E-09 |  | TNFSF15 | 0.376623312 | 6.63E-24 | NECTIN3 | 0.258273734 | 1.26E-11 |
| PDCD1LG2 | 0.440507288 | 4.92E-33 |  | CD48 | 0.359914385 | 7.88E-22 | BTN3A3 | 0.075587301 | 0.051024 |
| SIRPA | -0.10330433 | 0.007583 |  | LILRA6 | 0.450345 | 1.27E-34 | EDAR | -0.09914436 | 0.010406 |
| PDCD1 | 0.241648502 | 2.56E-10 |  | TNFRSF10A | 0.430757204 | 1.64E-31 | TNFRSF19 | 0.194077079 | 4.39E-07 |
| NECTIN1 | -0.28718744 | 3.94E-14 |  | VTCN1 | 0.116175238 | 0.002656 | TNFRSF13B | 0.063175745 | 0.103066 |
| ICOSLG | 0.163014966 | 2.33E-05 |  | ANGPTL7 | 0.013928583 | 0.719545 | TNFSF12 | 0.304416535 | 9.07E-16 |
| CD226 | 0.288007151 | 3.31E-14 |  | TNFSF13 | 0.369805462 | 4.82E-23 | SIRPB1 | 0.352388349 | 6.20E-21 |
| TNFRSF21 | -0.13277688 | 0.000586 |  | NGFR | 0.0806536 | 0.037299 | TNFRSF4 | 0.283232416 | 9.03E-14 |
| CD40 | 0.471805118 | 2.84E-38 |  | CD80 | 0.30160207 | 1.71E-15 | SLAMF7 | 0.252016855 | 4.02E-11 |
| TNFSF18 | -0.07282952 | 0.060123 |  | LY9 | 0.283702871 | 8.19E-14 | CD84 | 0.208335068 | 5.63E-08 |
| TNFSF11 | 0.402671834 | 2.17E-27 |  | TNFRSF9 | 0.465558431 | 3.49E-37 | FAS | 0.424259597 | 1.60E-30 |

**Supplementary Table S6. The clinical features of 26 GBM patients.**

| **Characteristic** |  | **Number of Glioma tissues** |
| --- | --- | --- |
| Age (years) | <=60 | 19 |
|  | >60 | 7 |
| Gender | Female | 15 |
|  | Male | 11 |
| WHO grade | II | 0 |
|  | III | 0 |
|  | IV | 26 |
| IDH Status | Mutant | 6 |
|  | Wild type | 20 |
| TMZ chemotherapy | Yes | 22 |
|  | No | 4 |
| Radiotherapy | Yes | 21 |
|  | No | 5 |

TMZ, Temozolomide.

**Supplementary Table S7. 119 differentially expressed genes between the low and medium infiltration group**

| Low and medium infiltration group | | | | | | | | | |
| --- | --- | --- | --- | --- | --- | --- | --- | --- | --- |
| Gene (Up) | logFC | logCPM | PValue | FDR | Gene (Down) | logFC | logCPM | PValue | FDR |
| TIMP1 | 3.459626 | 12.75745 | 2.64E-64 | 8.28E-62 | ADAMTS13 | -1.1319 | 8.644194 | 8.55E-35 | 1.43E-33 |
| S100A4 | 2.749783 | 10.06071 | 3.42E-64 | 8.28E-62 | PLXNB3 | -1.1432 | 10.55271 | 4.24E-33 | 6.23E-32 |
| ANXA2 | 2.216583 | 10.6169 | 1.96E-51 | 3.17E-49 | ADAM22 | -1.1221 | 10.14934 | 7.28E-32 | 1.04E-30 |
| SERPING1 | 1.840406 | 11.34269 | 9.72E-51 | 1.18E-48 | MST1 | -1.2427 | 9.013838 | 3.32E-30 | 4.11E-29 |
| S100A11 | 1.673595 | 12.89488 | 8.30E-48 | 8.05E-46 | NTNG2 | -1.1684 | 9.250005 | 8.29E-30 | 9.57E-29 |
| IGFBP2 | 3.254646 | 11.6933 | 2.04E-45 | 1.65E-43 | AMH | -1.3585 | 9.450966 | 2.30E-26 | 1.99E-25 |
| LGALS3 | 2.228669 | 11.47485 | 9.53E-45 | 6.60E-43 | PAPLN | -1.1607 | 8.865152 | 2.40E-23 | 1.73E-22 |
| POSTN | 5.521392 | 10.0079 | 3.46E-43 | 2.10E-41 | COL7A1 | -1.1913 | 8.385537 | 2.57E-23 | 1.84E-22 |
| PLAU | 2.334906 | 9.407946 | 8.28E-43 | 4.46E-41 | COL11A2 | -1.1130 | 8.759559 | 5.50E-22 | 3.51E-21 |
| MMP9 | 4.302863 | 9.275725 | 5.37E-42 | 2.61E-40 | BMP2 | -1.2770 | 10.69136 | 2.69E-18 | 1.36E-17 |
| COL3A1 | 3.057104 | 10.37292 | 2.99E-41 | 1.32E-39 | MEGF11 | -1.2020 | 9.659307 | 9.30E-18 | 4.51E-17 |
| CXCL10 | 3.350065 | 9.459832 | 5.58E-41 | 2.25E-39 | SLIT1 | -1.1403 | 11.34792 | 2.08E-17 | 9.15E-17 |
| COL1A1 | 2.918803 | 9.925612 | 8.02E-41 | 2.99E-39 | SEMA3D | -1.270 | 8.351546 | 5.30E-17 | 2.22E-16 |
| SERPINE1 | 2.652084 | 10.88843 | 1.30E-40 | 4.51E-39 | HAPLN1 | -1.2453 | 9.157832 | 2.40E-16 | 9.32E-16 |
| COL4A1 | 2.339746 | 10.97772 | 5.97E-40 | 1.93E-38 | THSD4 | -1.1527 | 8.132573 | 4.26E-16 | 1.62E-15 |
| SERPINH1 | 1.63956 | 10.42961 | 9.36E-40 | 2.84E-38 | WNT7B | -1.3073 | 9.191925 | 1.86E-15 | 6.77E-15 |
| ANXA2P2 | 2.871492 | 8.647606 | 6.17E-39 | 1.76E-37 | TNR | -1.2806 | 12.38151 | 2.40E-15 | 8.62E-15 |
| COL6A2 | 2.350857 | 10.44452 | 5.45E-38 | 1.47E-36 | SMOC1 | -1.3254 | 13.59886 | 9.18E-15 | 3.18E-14 |
| S100A9 | 2.416996 | 11.08963 | 6.20E-38 | 1.58E-36 | FGF17 | -1.2327 | 8.137913 | 1.14E-13 | 3.46E-13 |
| LGALS1 | 1.447631 | 13.70187 | 1.20E-37 | 2.91E-36 | SFRP2 | -1.5710 | 12.09845 | 9.73E-13 | 2.73E-12 |
| COL1A2 | 2.159186 | 10.07639 | 2.64E-37 | 6.09E-36 | CBLN2 | -1.1414 | 8.206433 | 2.06E-09 | 4.81E-09 |
| IBSP | 4.500294 | 8.719451 | 4.18E-37 | 9.21E-36 | HPSE2 | -1.256 | 9.438328 | 2.08E-09 | 4.82E-09 |
| FMOD | 3.458787 | 10.42074 | 4.86E-37 | 1.03E-35 |  |  |  |  |  |
| COL4A2 | 1.981563 | 10.99622 | 7.47E-37 | 1.51E-35 |  |  |  |  |  |
| MGP | 2.420366 | 11.07792 | 1.49E-36 | 2.89E-35 |  |  |  |  |  |
| ANXA1 | 2.308302 | 11.44719 | 1.93E-36 | 3.60E-35 |  |  |  |  |  |
| SERPINA1 | 1.822363 | 9.548978 | 4.17E-36 | 7.49E-35 |  |  |  |  |  |
| SRPX2 | 2.321672 | 9.126853 | 3.73E-35 | 6.46E-34 |  |  |  |  |  |
| CCL5 | 1.81498 | 8.506011 | 1.53E-34 | 2.47E-33 |  |  |  |  |  |
| FSTL1 | 1.336625 | 10.50745 | 2.37E-34 | 3.71E-33 |  |  |  |  |  |
| GDF15 | 2.910004 | 8.66771 | 1.28E-33 | 1.94E-32 |  |  |  |  |  |
| F13A1 | 2.619622 | 9.387278 | 1.05E-31 | 1.46E-30 |  |  |  |  |  |
| TNFAIP6 | 2.414658 | 8.93714 | 2.21E-31 | 2.98E-30 |  |  |  |  |  |
| PLAT | 1.944379 | 9.763662 | 4.81E-31 | 6.30E-30 |  |  |  |  |  |
| LUM | 2.082479 | 8.707222 | 5.14E-30 | 6.08E-29 |  |  |  |  |  |
| S100A6 | 1.260382 | 14.59105 | 4.12E-29 | 4.64E-28 |  |  |  |  |  |
| PI3 | 4.24069 | 9.292652 | 1.41E-28 | 1.55E-27 |  |  |  |  |  |
| SERPINA5 | 3.077274 | 8.285538 | 1.45E-28 | 1.56E-27 |  |  |  |  |  |
| MMP14 | 1.300297 | 11.11253 | 1.64E-28 | 1.72E-27 |  |  |  |  |  |
| CLEC5A | 2.473752 | 8.183593 | 1.94E-28 | 2.01E-27 |  |  |  |  |  |
| FBLN5 | 1.398284 | 9.285394 | 3.14E-28 | 3.17E-27 |  |  |  |  |  |
| LIF | 2.631358 | 8.386182 | 8.04E-28 | 7.96E-27 |  |  |  |  |  |
| SPP1 | 1.761708 | 15.51427 | 1.62E-27 | 1.57E-26 |  |  |  |  |  |
| VEGFA | 1.952889 | 10.04269 | 2.57E-27 | 2.45E-26 |  |  |  |  |  |
| PCOLCE | 1.806113 | 9.498866 | 5.28E-27 | 4.83E-26 |  |  |  |  |  |
| S100A8 | 2.091089 | 9.489152 | 7.40E-27 | 6.65E-26 |  |  |  |  |  |
| FN1 | 1.182589 | 11.53317 | 1.12E-26 | 9.85E-26 |  |  |  |  |  |
| EFEMP2 | 1.344464 | 9.889262 | 2.96E-26 | 2.51E-25 |  |  |  |  |  |
| LOX | 2.189981 | 8.610196 | 1.63E-25 | 1.34E-24 |  |  |  |  |  |
| S100A10 | 1.32503 | 12.51799 | 2.55E-24 | 2.02E-23 |  |  |  |  |  |
| ESM1 | 2.733489 | 8.306892 | 2.78E-24 | 2.18E-23 |  |  |  |  |  |
| CLCF1 | 1.9236 | 8.373534 | 3.42E-24 | 2.63E-23 |  |  |  |  |  |
| COL5A2 | 1.555192 | 9.541125 | 1.40E-23 | 1.04E-22 |  |  |  |  |  |
| COL8A1 | 2.496729 | 8.210324 | 1.57E-23 | 1.15E-22 |  |  |  |  |  |
| LAMB1 | 1.435668 | 9.438571 | 3.32E-23 | 2.33E-22 |  |  |  |  |  |
| ADAM12 | 2.060276 | 8.112081 | 4.58E-23 | 3.17E-22 |  |  |  |  |  |
| TGFBI | 1.627192 | 10.01005 | 5.76E-23 | 3.94E-22 |  |  |  |  |  |
| CSTA | 1.889302 | 8.037456 | 1.03E-22 | 6.78E-22 |  |  |  |  |  |
| SFRP4 | 1.589001 | 9.409632 | 1.18E-22 | 7.66E-22 |  |  |  |  |  |
| CCL2 | 1.716149 | 10.75534 | 6.21E-22 | 3.91E-21 |  |  |  |  |  |
| SERPINA3 | 1.914884 | 8.130818 | 2.97E-21 | 1.84E-20 |  |  |  |  |  |
| TGFB2 | 1.465106 | 9.660063 | 4.23E-21 | 2.57E-20 |  |  |  |  |  |
| CXCL11 | 2.344777 | 8.057731 | 4.47E-21 | 2.68E-20 |  |  |  |  |  |
| CTHRC1 | 1.911722 | 8.758562 | 1.70E-20 | 9.93E-20 |  |  |  |  |  |
| COL5A1 | 1.91344 | 8.50095 | 1.88E-20 | 1.09E-19 |  |  |  |  |  |
| LOXL2 | 1.332753 | 9.106075 | 3.12E-20 | 1.76E-19 |  |  |  |  |  |
| IGFBP3 | 1.665054 | 11.37768 | 3.44E-20 | 1.92E-19 |  |  |  |  |  |
| ANGPT2 | 1.432961 | 8.844982 | 5.66E-20 | 3.12E-19 |  |  |  |  |  |
| THBS1 | 1.759964 | 8.762989 | 1.85E-19 | 9.69E-19 |  |  |  |  |  |
| HTRA3 | 1.759042 | 8.063349 | 1.86E-19 | 9.69E-19 |  |  |  |  |  |
| AEBP1 | 1.476536 | 12.07817 | 2.30E-19 | 1.19E-18 |  |  |  |  |  |
| EMILIN2 | 1.417765 | 8.393722 | 3.56E-18 | 1.77E-17 |  |  |  |  |  |
| S100A3 | 1.574679 | 8.815688 | 5.17E-18 | 2.53E-17 |  |  |  |  |  |
| ST14 | 1.193204 | 8.484791 | 1.06E-17 | 5.11E-17 |  |  |  |  |  |
| FRZB | 1.138118 | 9.503518 | 1.35E-17 | 6.35E-17 |  |  |  |  |  |
| SERPINF1 | 1.358688 | 10.33259 | 1.35E-17 | 6.35E-17 |  |  |  |  |  |
| LOXL1 | 1.579235 | 8.303245 | 1.59E-17 | 7.33E-17 |  |  |  |  |  |
| MXRA5 | 1.644234 | 8.196048 | 2.31E-17 | 1.01E-16 |  |  |  |  |  |
| CLEC2B | 1.182821 | 8.332988 | 5.86E-17 | 2.43E-16 |  |  |  |  |  |
| CLEC18B | 1.51041 | 8.081457 | 3.02E-16 | 1.15E-15 |  |  |  |  |  |
| SDC1 | 1.345247 | 8.69856 | 1.49E-15 | 5.48E-15 |  |  |  |  |  |
| WISP1 | 1.92884 | 8.173275 | 4.22E-15 | 1.48E-14 |  |  |  |  |  |
| MMP7 | 2.340249 | 8.130886 | 9.38E-15 | 3.23E-14 |  |  |  |  |  |
| SPON2 | 1.471122 | 8.096997 | 1.60E-14 | 5.42E-14 |  |  |  |  |  |
| MFAP2 | 1.862326 | 8.255324 | 2.63E-14 | 8.73E-14 |  |  |  |  |  |
| HSPG2 | 1.102859 | 8.653817 | 4.55E-14 | 1.44E-13 |  |  |  |  |  |
| NPNT | 1.371788 | 9.300706 | 5.33E-14 | 1.65E-13 |  |  |  |  |  |
| SEMA3F | 1.33908 | 8.302044 | 5.39E-14 | 1.65E-13 |  |  |  |  |  |
| ADAM33 | 1.477499 | 8.29968 | 1.72E-13 | 5.05E-13 |  |  |  |  |  |
| SLPI | 1.382337 | 10.39307 | 8.06E-13 | 2.29E-12 |  |  |  |  |  |
| IL6 | 1.602981 | 7.985835 | 6.88E-11 | 1.79E-10 |  |  |  |  |  |
| FBLN7 | 1.160355 | 8.071187 | 6.99E-11 | 1.81E-10 |  |  |  |  |  |
| SFRP5 | 2.17176 | 8.397441 | 1.90E-10 | 4.82E-10 |  |  |  |  |  |
| COL14A1 | 1.181789 | 8.306523 | 2.19E-10 | 5.54E-10 |  |  |  |  |  |
| EMILIN3 | 1.391127 | 9.425264 | 2.03E-09 | 4.77E-09 |  |  |  |  |  |
| IGF2 | 1.317867 | 10.25596 | 3.00E-09 | 6.90E-09 |  |  |  |  |  |
| FGFBP2 | 1.106011 | 8.504157 | 2.73E-07 | 5.52E-07 |  |  |  |  |  |

Log FC, log_2_ (Fold change); log CPM, log_2_ (Counts er million), FDR, False discovery rate.

**Supplementary Table S8. 76 differentially expressed between the medium and high infiltration groups**

| Medium and high infiltration groups | | | | | | | | | |
| --- | --- | --- | --- | --- | --- | --- | --- | --- | --- |
| Gene (Up) | logFC | logCPM | PValue | FDR | Gene (Down) | logFC | logCPM | PValue | FDR |
| CCL18 | 4.474854 | 8.854269 | 3.54E-53 | 1.79E-50 | SEMA6C | -1.1123 | 9.043892 | 7.52E-11 | 8.10E-10 |
| LUM | 2.673902 | 9.456321 | 2.53E-51 | 6.40E-49 | COL11A2 | -1.3716 | 8.349853 | 1.91E-10 | 1.85E-09 |
| FGFBP2 | 3.376213 | 9.415564 | 5.38E-44 | 9.08E-42 | SLIT1 | -1.5090 | 10.81799 | 2.07E-09 | 1.63E-08 |
| MMP13 | 4.110542 | 7.698463 | 1.76E-42 | 2.22E-40 | NTNG2 | -1.2207 | 8.807038 | 6.69E-09 | 4.91E-08 |
| COL6A3 | 2.805896 | 8.414699 | 4.55E-39 | 4.60E-37 | MMP24 | -1.2279 | 9.073876 | 8.65E-09 | 6.25E-08 |
| COL1A2 | 2.346691 | 10.91809 | 1.30E-37 | 1.10E-35 | GPC2 | -1.1351 | 9.14112 | 1.49E-08 | 1.03E-07 |
| CXCL9 | 2.244476 | 8.159775 | 1.10E-36 | 7.97E-35 | HAPLN2 | -1.5067 | 10.1512 | 1.88E-08 | 1.27E-07 |
| CXCL13 | 3.353932 | 8.140133 | 1.39E-34 | 8.76E-33 | ADAM11 | -1.4500 | 8.615833 | 3.98E-08 | 2.58E-07 |
| COL1A1 | 2.591583 | 10.88718 | 2.79E-34 | 1.57E-32 | CBLN1 | -1.8328 | 8.377551 | 4.08E-08 | 2.61E-07 |
| COL3A1 | 2.594428 | 11.38172 | 3.63E-31 | 1.84E-29 | SFRP5 | -2.9379 | 8.401536 | 7.80E-08 | 4.64E-07 |
| CTSC | 1.180385 | 9.213676 | 2.20E-30 | 1.01E-28 | FGFBP3 | -1.1758 | 9.697533 | 1.05E-07 | 5.88E-07 |
| CCL5 | 1.350246 | 8.787996 | 1.03E-28 | 4.36E-27 | VWA5B2 | -1.3308 | 8.025202 | 1.92E-07 | 9.95E-07 |
| F13A1 | 2.273885 | 10.12138 | 2.26E-25 | 8.81E-24 | AMH | -1.1305 | 8.912306 | 3.69E-07 | 1.79E-06 |
| CST7 | 1.27161 | 7.983418 | 4.98E-23 | 1.80E-21 | C1QL2 | -1.5326 | 8.24386 | 4.63E-07 | 2.17E-06 |
| LTBP2 | 1.199201 | 8.335466 | 2.37E-21 | 7.50E-20 | NTN4 | -1.1919 | 9.199338 | 2.06E-06 | 8.52E-06 |
| MMP19 | 1.595988 | 8.07103 | 8.22E-20 | 2.45E-18 | MEGF11 | -1.1627 | 9.181284 | 3.03E-06 | 1.17E-05 |
| COL6A2 | 1.743743 | 11.09061 | 2.11E-18 | 5.63E-17 | CRHBP | -1.1922 | 8.048721 | 3.19E-06 | 1.21E-05 |
| CSTA | 1.142973 | 8.143768 | 2.21E-17 | 5.58E-16 | TNR | -1.4199 | 11.80123 | 3.88E-06 | 1.40E-05 |
| THBS1 | 1.609259 | 9.128336 | 1.07E-16 | 2.46E-15 | LGI3 | -1.1587 | 10.01161 | 1.15E-05 | 3.79E-05 |
| S100A9 | 1.610829 | 11.71537 | 7.36E-16 | 1.55E-14 | SPOCK3 | -1.1582 | 9.681826 | 1.48E-05 | 4.70E-05 |
| PCOLCE | 1.497538 | 9.960918 | 2.80E-15 | 5.45E-14 | COL28A1 | -1.2142 | 8.653894 | 2.48E-05 | 7.42E-05 |
| IGFBP6 | 1.330435 | 9.62371 | 3.07E-15 | 5.76E-14 | C1QL4 | -1.2847 | 8.888664 | 4.23E-05 | 0.000123 |
| S100A8 | 1.592832 | 9.978245 | 4.32E-15 | 7.81E-14 | COL20A1 | -1.2034 | 9.691024 | 6.13E-05 | 0.000174 |
| COL15A1 | 1.267046 | 7.84053 | 5.33E-15 | 9.30E-14 | NELL1 | -1.2693 | 7.939873 | 6.59E-05 | 0.000184 |
| COL5A1 | 1.385471 | 8.767572 | 1.32E-12 | 1.97E-11 | CLEC2L | -1.3289 | 8.183775 | 6.99E-05 | 0.000193 |
| ASPN | 1.316732 | 8.025685 | 1.48E-12 | 2.15E-11 | RELN | -1.1718 | 7.956739 | 0.000184 | 0.00046 |
| S100A4 | 1.144321 | 10.53462 | 3.07E-12 | 4.32E-11 | WIF1 | -1.6586 | 8.560359 | 0.000219 | 0.00054 |
| PI3 | 2.345003 | 10.05665 | 7.43E-12 | 9.90E-11 | WNT7B | -1.1003 | 8.712867 | 0.00022 | 0.00054 |
| TGFBI | 1.214284 | 10.42043 | 9.16E-12 | 1.19E-10 | SMOC1 | -1.1545 | 12.99268 | 0.000273 | 0.000642 |
| ADAMDEC1 | 1.492616 | 7.938456 | 3.41E-11 | 4.02E-10 | C1QL3 | -1.4331 | 8.13592 | 0.000346 | 0.0008 |
| SERPINE1 | 1.413032 | 11.46125 | 3.90E-11 | 4.38E-10 |  |  |  |  |  |
| CTHRC1 | 1.342083 | 9.074342 | 1.34E-10 | 1.42E-09 |  |  |  |  |  |
| CXCL5 | 1.352772 | 8.199541 | 1.90E-10 | 1.85E-09 |  |  |  |  |  |
| SRPX2 | 1.19717 | 9.492194 | 1.97E-10 | 1.88E-09 |  |  |  |  |  |
| IL1RN | 1.161859 | 7.823471 | 2.30E-10 | 2.16E-09 |  |  |  |  |  |
| LOX | 1.2965 | 8.896507 | 2.60E-10 | 2.35E-09 |  |  |  |  |  |
| TIMP1 | 1.330708 | 13.39354 | 3.47E-10 | 3.08E-09 |  |  |  |  |  |
| LIF | 1.288902 | 8.629712 | 2.28E-09 | 1.77E-08 |  |  |  |  |  |
| CXCL10 | 1.412657 | 9.974444 | 3.07E-09 | 2.35E-08 |  |  |  |  |  |
| IL6 | 1.310973 | 8.066837 | 9.42E-09 | 6.65E-08 |  |  |  |  |  |
| CXCL1 | 1.27548 | 8.279277 | 1.56E-08 | 1.07E-07 |  |  |  |  |  |
| CXCL14 | 1.194457 | 11.48124 | 4.37E-08 | 2.73E-07 |  |  |  |  |  |
| IBSP | 1.595902 | 9.126226 | 6.34E-08 | 3.82E-07 |  |  |  |  |  |
| OGN | 1.154899 | 8.186144 | 3.57E-06 | 1.33E-05 |  |  |  |  |  |
| POSTN | 1.507065 | 10.61784 | 1.13E-05 | 3.75E-05 |  |  |  |  |  |
| MMP9 | 1.116828 | 9.670907 | 0.000116 | 0.000307 |  |  |  |  |  |

Log FC, log_2_ (Fold change); log CPM, log_2_ (Counts er million), FDR, False discovery rate.

**Supplementary Table S9. 36 matisomes in Lasso analysis.**

| Gene (Up) (n=28) | Gene (Down) (n=8) |
| --- | --- |
| LUM | NTNG2 |
| FGFBP2 | AMH |
| COL1A2 | COL11A2 |
| COL1A1 | MEGF11 |
| COL3A1 | SLIT1 |
| CCL5 | WNT7B |
| F13A1 | TNR |
| COL6A2 | SMOC1 |
| CSTA |  |
| THBS1 |  |
| S100A9 |  |
| PCOLCE |  |
| S100A8 |  |
| COL5A1 |  |
| S100A4 |  |
| PI3 |  |
| TGFBI |  |
| SERPINE1 |  |
| CTHRC1 |  |
| SRPX2 |  |
| LOX |  |
| TIMP1 |  |
| LIF |  |
| CXCL10 |  |
| IL6 |  |
| IBSP |  |
| POSTN |  |
| MMP9 |  |

**Supplementary Table S10. The comparison of clinicopathological characteristics between the high-risk and low-risk groups in the GEO database.**

| **Clinicopathological characteristics** | **High-risk group**  **(n = 169)** | **Low-risk group**  **(n = 170)** | ***p-*value** |
| --- | --- | --- | --- |
| **Age** (Mean ± SD) | 58.23±9.84 | 54.86±11.01 | <0.001 |
| **Gender** |  |  | 0.831 |
| Male | 109 (64.5%) | 109 (64.1%) |  |
| Female | 60 (35.5%) | 61 (35.9%) |  |
| **WHO grade** |  |  | <0.001 |
| II | 25 (14.8%) | 33 (19.4%) | <0.001 |
| III | 33 (19.5%) | 71 (41.8%) |  |
| IV | 111 (65.7%) | 66 (38.8%) |  |
| **Karnofsky Performance Score** |  |  |  |
| 50-80 | 56 (33.1%) | 93 (54.7%) |  |
| 90-100 | 113 (66.9%) | 77 (45.3%) |  |
| **Mini-mental State Examination** |  |  | <0.001 |
| <27 | 108(63.9%) | 62 (36.5%) |  |
| >=27 | 61 (36.1%) | 108 (63.5%) |  |
| **MGMT promoter status** |  |  | <0.001 |
| Methylated | 66 (39.1%) | 116 (68.2%) |  |
| Unmethylated | 103 (60.9%) | 54 (31.8%) |  |
|  |  |  |  |
